# Why researchers cut corners: motivations, pressures, and personality in questionable research practices

**DOI:** 10.1186/s41073-026-00229-8

**Published:** 2026-07-17

**Authors:** Robin Brooker

**Affiliations:** School of Law, University of Reading, Reading, UK

**Keywords:** Questionable Research Practices (QRPs), Research integrity, Mertonian norms, Publication, Pressure, Personality traits, Motivations

## Abstract

**Background:**

Some questionable research practices (QRPs) are regarded as core contributors to problems of reproducibility and replication, while others compromise the integrity and credibility of research by obscuring responsibility and incentives. However, evidence on the individual-level drivers of QRP engagement is fragmented. This study examines theory-informed associations between social and psychological factors and self-reported QRP engagement.

**Methods:**

Using data from a cross-sectional survey of researchers across disciplinary fields (18,376 invited; N = 3,050 respondents), this study examines associations between social and psychological factors and self-reported QRP engagement. The sample reflects a self-selected subset of invited respondents.

**Results:**

I found that commitment to universalism (β = -0.14, 95% CI [-0.18, -0.10]), communalism (β = -0.04, 95% CI [-0.08, 0.0]), and organised scepticism (β = -0.15, 95% CI [-0.2, -0.1]) – as well as higher conscientiousness (β = -0.07, 95% CI [-0.11, -0.03]), agreeableness (β = -0.03, 95% CI [-0.07, 0.00]), emotional stability (opposite to neuroticism) (β = -0.07, 95% CI [-0.11, -0.04]), and openness (β = -0.08, 95% CI [-0.12, -0.04]) – were negatively associated with average self-reported QRP engagement. Publication pressure was associated with greater average self-reported QRP engagement (β = 0.12, 95% CI [0.08, 0.16]), but this association was weaker among respondents with higher scores on a factor interpreted as endorsement of universalism (β = -0.04, 95% CI [-0.08, 0.00]). Funding pressure was not statistically associated with self-reported average QRP engagement. Motivations to falsify theories and findings (β = -0.06, 95% CI [-0.11, -0.01]), and to improve impact and quality of results (β = -0.11, 95% CI [-0.15, -0.06]), were negatively associated with self-reported QRP engagement. Conversely, ego and esteem-related motivation (e.g., fame, achieving social and political goals) was found to have a direct association with mean self-reported QRP engagement (β = 0.11, 95% CI [0.07, 0.15]), whereas career-growth motivations showed no clear direct association (β = -0.03, 95% CI [-0.07, 0.01]). However, both were indirectly associated with QRP engagement through scientific achievement and impact/quality motivations.

**Conclusions:**

These findings highlight specific social and psychological factors that are associated with self-reported QRP engagement, offering potential leverage points for efforts to promote research integrity and reduce questionable practices. However, because the analytic sample reflects a self-selected subset of invited researchers and is drawn largely from Europe and the United States, the findings may not fully generalise to the broader global research community.

**Supplementary Information:**

The online version contains supplementary material available at https://doi.org/10.1186/s41073-026-00229-8.

## Introduction

Questionable research practices (QRPs) can undermine the validity, reliability, and reproducibility of scientific findings, potentially compromising the integrity of the evidence base and, in turn, the quality of decisions and interventions based upon it. QRPs are without straightforward definition, but exist in an area of ethical ambiguity between outright misconduct (i.e., falsification, fabrication and plagiarism) and sound scientific practice. These practices involve the exploitation of the inherent flexibility in the research process and may be perceived as warranted or defensible in certain contexts [1–3]. QRPs are widely discussed as possible contributors to issues of scientific replicability and reproducibility ([1, 4–6]; cf. [7]), although interpretations of replication outcomes and the extent to which this constitutes a ‘replication crisis’ are contested (see, [8–10]). Beyond replicability, QRPs can undermine transparency and accountability, and weaken evidence synthesis. Estimates of QRP prevalence vary considerably across studies, though most self-report surveys and meta-analyses place prevalence levels between 12.5% and 34% [11–16].^1^ Given their potential impact, it is important to identify factors associated with engagement in these behaviours and to evaluate theoretically proposed pathways linking them.

The preeminent theory is that QRPs result from a misalignment between incentive structures and the ideals of good scientific practice. Academic institutions often use simple metrics, such as frequency of publication, journal impact factors and citation counts when assessing for hiring, permanency, tenure and promotion [17]. The theory suggests that these systemic factors fuel hyper-competition and perceived pressure, which in turn motivate researchers to cut corners to maximise their chances of publishing in high-impact journals [12, 18, 19]. Evidence supporting this ‘publish and perish’ argument is derived from correlational research that finds self-reported publication pressure to be positively associated with self-reported QRP engagement [14, 15, 20]. However, the observed effect sizes remain small.

The influence of social and psychological factors on QRP engagement remains largely unexplored. The limited research on individual differences suggests that these factors are at least as important as system-level influences in explaining QRP engagement [14, 15, 20]. For example, correlational research suggests that the personality traits of agreeableness and conscientiousness are negatively associated with self-reported engagement in QRPs [15, 21]. Moreover, a researcher’s commitment to the normative ideals of science theorised by Robert Merton^2^ [22], have been found to be negatively associated with self-reported QRP engagement [14, 20]. Adherence to these normative ideals has been found to be widespread, but not universal [23–26]. These norms may reduce susceptibility to QRPs by functioning as internalised standards of appropriate scientific conduct, thereby constraining behaviour even when external incentives encourage productivity, novelty, or positive results. In this sense, commitment to scientific norms may serve as a counterweight to pressures that reward strategic or outcome-oriented research behaviour. The limited research examining the role of these scientific norms in relation to QRP engagement suggests that commitment to these ideals is among the strongest correlates of self-reported QRP engagement [14, 20]. However, in the studies conducted to date, scientific norms have been treated as a single, unified construct. As a result, the potentially distinct influence of each of the Mertonian norms remains largely unexplored in the existing literature.

Another potentially important yet underexamined individual-level factor is scientific orientation – namely, the extent to which researchers are motivated to falsify versus confirm hypotheses, findings and theories. A falsification-oriented approach, rooted in Popperian ideals of critical testing, may discourage selective reporting and p-hacking by orienting researchers towards attempts to challenge rather than protect their preferred hypotheses. By contrast, a confirmatory orientation may increase susceptibility to QRPs in so far that it encourages practices to preserve, strengthen or selectively present results consistent with an expected outcome and thereby increase their publishability. This relationship has received limited empirical attention, yet any meaningful discussion of the influence of epistemic norms on research practice must also account for how scientists approach the research process.

Jussim et al. [27] proposed that both personal motivations (e.g., career advancement) and proximal science-specific motivations (e.g., increasing the publication frequency) may influence a researcher’s propensity to engage in QRPs. Researchers motivated by personal gain – such as wealth, recognition, or prestige – may be more vulnerable to compromising scientific integrity in pursuit of these ends. When career-relevant rewards depend heavily on producing novel, positive, or high-impact findings, personal motivations such as career ambition, recognition, or esteem may increase willingness to engage in selective reporting or other forms of strategic research behaviour that improve the likelihood of desirable outcomes. Proximal science-specific motivations are likely to matter because they shape the immediate goals researchers pursue in their day-to-day work. When these goals emphasise productivity, visibility, or external markers of success, researchers may become more willing to use QRPs to obtain publishable results. By contrast, motivations centred on improving research quality and substantive impact may discourage QRPs by orienting researchers towards accuracy, robustness, and the long-term value of their findings.

Jussim and colleagues [27] further speculate that system-level factors, such as unequal incentive structures, may moderate the relationship between personal motivations and proximal science-specific motivations. Institutional incentive structures may shape which routes for pursuing personal motivations appear most instrumentally useful within scientific work. In settings where publication counts, grant capture, and high-impact outputs are strongly rewarded, personal motivations such as career ambition or ego and esteem may be more readily channelled into proximal science-specific motivations, such as increasing publication frequency or maximising research impact. By contrast, where such incentives are weaker or less salient, the same personal motivations may be less strongly translated into science-specific motivations. On this view, publication and funding pressure do not merely exert direct effects on QRP engagement, but may condition the extent to which underlying personal motivations are expressed through more proximal scientific motivations.

Existing evidence explaining why researchers engage in QRPs is fragmented, typically focusing on single predictors or treating key constructs (e.g., scientific norms) as unitary. I extend this literature by conducting a series of theory-informed analyses that (a) disaggregates Mertonian norms, (b) incorporates scientific orientation and motivations alongside systemic pressures, and (c) tests moderation and mediation pathways.

### The current study

In this study, I use data from an international survey of researchers operating across disciplinary fields and countries to empirically test theory-informed associations among motivational, normative, individual-difference, and systemic predictors of self-reported QRP engagement. The study seeks to build on existing fragmentary evidence by integrating these predictors within a single theory-informed framework.

I partially build on the theoretical model developed by Jussim et al. [27] to examine the extent to which personal motivations and proximal science-specific motivations are associated with self-reported QRP engagement. I hypothesise that individuals more strongly motivated by career achievement (H1) and ego and esteem (H2) will report greater QRP engagement. I further hypothesise that scientific achievement motivation will be positively associated with self-reported QRP engagement (H3), whereas motivation to increase the impact and quality of one’s research will be negatively associated with self-reported QRP engagement (H4). As proposed by Jussim et al. [27], personal motivations may influence self-reported QRP engagement indirectly through more proximal science-specific motivations. Jussim et al. distinguish broader personal motivations (e.g., status, recognition, career advancement) from more proximal science-specific motivations (e.g., publishing, grant acquisition, producing high-impact findings), and propose that the former shape the latter within institutional reward structures. The theoretical rationale for this ordering is that personal motivations describe broad desired outcomes that are not inherently tied to any specific research behaviour, whereas science-specific motivations describe more immediate goals within research practice and are therefore closer to the behavioural choices through which QRPs may occur. On this basis, I hypothesise that proximal science-specific motivations mediate the relationship between personal motivations and self-reported QRP engagement (H5-H8). Because the present analyses are based on cross-sectional correlational data, the mediation results should be interpreted as theory-informed indirect associations rather than definitive evidence of causal sequencing.

I hypothesise that self-reported publication pressure (H9) and funding pressure (H10) are positively associated with self-reported QRP engagement. Although these relationships feature prominently in theoretical accounts, with the influence of publication pressure receiving some empirical support, the evidence remains limited. This study examines these associations within a broader conceptual framework to assess their consistency and explanatory value. I further hypothesise that publication pressure (H11-H14) and funding pressure (H15-H18) moderate the relationship between personal motivations and science-specific motivations. These hypotheses follow from a translation account of motivation: broad personal motivations, such as career achievement, can be pursued through multiple routes, and institutional pressures may shape which routes appear most instrumentally useful. When publication and funding pressures are high, researchers may perceive publication frequency, grant success, and visible research impact as important means through which broader personal goals can be achieved. Under these conditions, personal motivations should be more strongly translated into proximal science-specific motivations centred on research productivity, visibility, and external success. Thus, the proposed moderation effect is not simply that publication or funding pressure directly increases science-specific motivations, but that these pressures condition the extent to which broader personal motives become expressed through scientific goals.

**Fig. 1:**
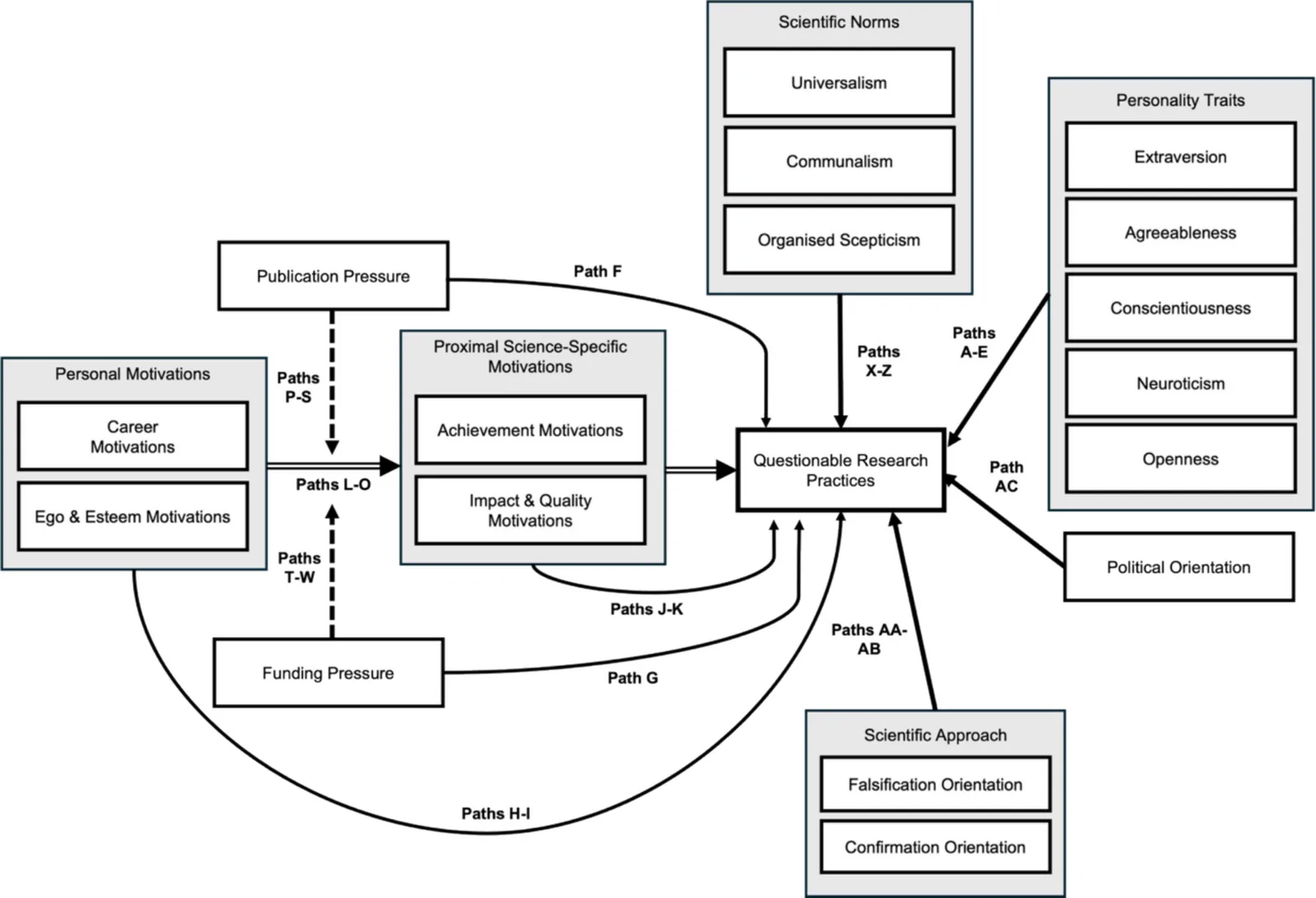
Conceptual framework illustrating potential social-psychological predictors of engagement in questionable research practices (QRPs). Note. The figure is intended to serve as a visual representation of the hypotheses and should not be construed as an overview of the analytical strategy. The compound (double-lined) arrow represents four mediation paths. The dashed-lines represent proposed moderating factors influencing the relationship between personal motivations and proximal science-specific motivations

Building on Schneider and colleagues' [15] findings that conscientiousness and agreeableness are negatively correlated with self-reported QRP engagement, I hypothesise that these personality traits are negatively associated with self-reported QRP engagement (H19a and H19b). I include all the big five personality traits, including emotional stability (opposite to neuroticism), openness and extraversion, in the model for completeness. Turning to other individual level factors, I explore whether commitment to the Mertonian norms of universalism, communalism, and organised scepticism differentially impact self-reported QRP engagement. Moreover, I include several additional exploratory elements. I explore whether a researcher's epistemic approach – specifically, whether they primarily seek to confirm findings/theories or adopt a Popperian falsification strategy – influences the extent of their self-reported engagement in QRPs. I also include a measure of political orientation (left–right ideological leaning) as an exploratory correlate of self-reported engagement in QRPs. Finally, as an additional exploratory element, I include several interaction terms to identify whether publication and funding pressure moderate the influence of scientific norms and personality traits on self-reported QRP engagement. Figure 1 presents the proposed theoretical framework.

The final manuscript does not reproduce the preregistered hypothesis structure verbatim. In particular, some preregistered motivational constructs were consolidated during scale development and factor analysis, some preregistered analyses were not retained in the final main-text presentation, and several additional analyses were conducted exploratorily. The preregistered confirmatory elements concerned publication and funding pressure, personal and proximal motivations, mediation and moderation pathways, and selected personality traits. Analyses involving the disaggregated Mertonian norms, epistemic orientation, political orientation, and additional interaction terms were treated as exploratory or as extensions beyond the preregistered framework where applicable. A full mapping of preregistered hypotheses to reported analyses and outcomes is provided in Additional File 1, Table S1.8.

Overall, the present study should be understood as an empirical test of a theory-informed associative framework rather than as a definitive test of causal mechanisms. Although the model is motivated by theoretical claims about how broader personal motivations, proximal science-specific motivations, and systemic pressures may relate to QRP engagement, the cross-sectional design cannot establish temporal ordering or causal mediation. The aim is therefore to evaluate whether the observed pattern of associations is consistent with the proposed framework and to identify which elements of that framework are supported by the data.

## Data and methods

### Sample

**Fig. 2:**
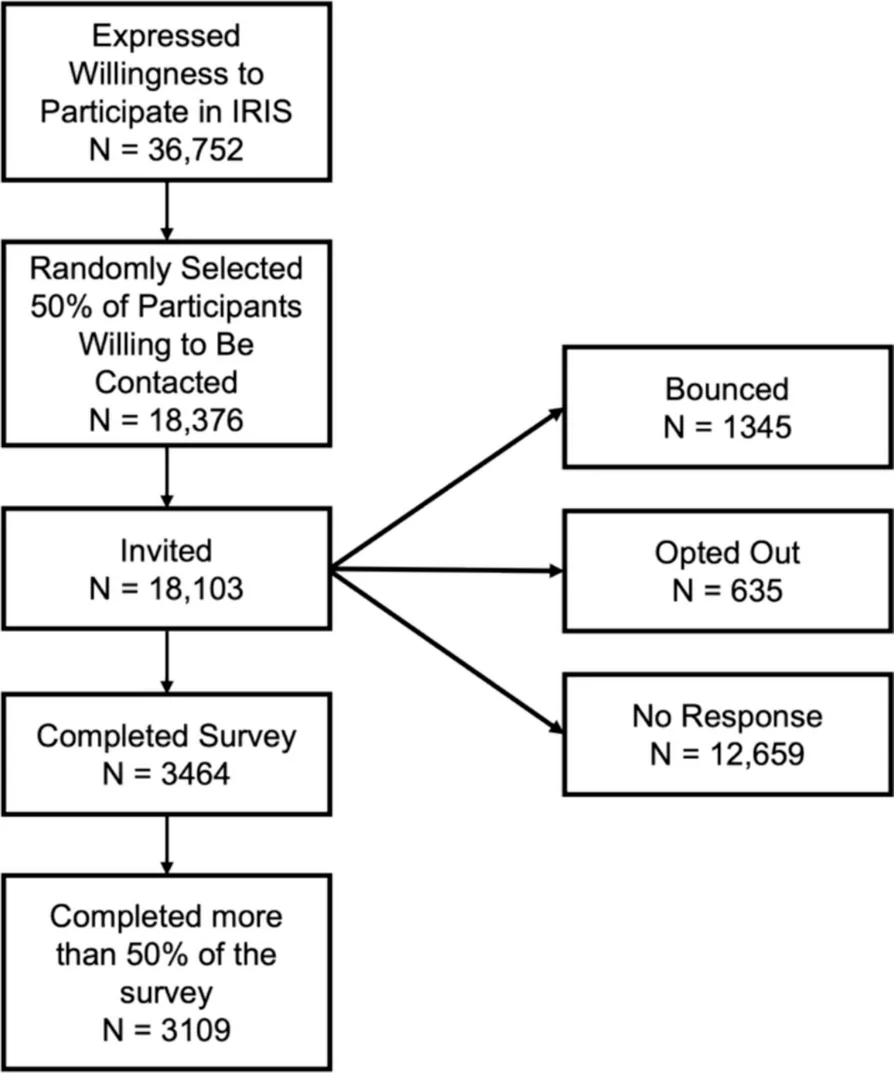
Flow chart of the international survey on research culture and integrity

The sample was derived from a list of respondents who indicated their willingness to participate in future research after completing IRIS [16]. Of those who indicated their willingness to participate in future studies, I randomly sampled 50% (*N* = 18,376). On this basis, the sample is a self-selected, non-probability volunteer sample. Weights were used to mitigate differential selection and nonresponse. The survey protocol was pre-registered with the Centre for Open Science in November 2024 (available, along with reproducibility materials, at [28]). I excluded individuals who had completed less than 50% of the survey (N = 355). The overall survey response rate was 19.3%, calculated using the American Association for Public Opinion Research’s standard definition [29], resulting in 3109 valid survey responses (see Fig. 2). I additionally removed respondents list-wise (*N* = 21) who answered fewer than four QRP items, before deriving the dependent variables. This reduced the analytical sample to 3088 respondents. Moreover, those selecting ‘prefer not to say’ (*N* = 38) to the gender item were removed list-wise from the analysis. This reduced the analytical sample to 3050.

### Study design

The survey was a cross-sectional survey on research integrity, academic culture and academic freedom, fielded between November and December 2024. It was conducted entirely in English and was initially piloted with twenty-five researchers in October and November 2024. The final responses were merged with IRIS [16], prior to being anonymised. Fully informed consent was obtained from all respondents, and ethical approval was given by the Ethics Committee in the Department of Sociology and Criminology at the University of Essex (approval number: ETH2324-0515).

### Survey instrument

The survey included sections on demographic measures, scientific values and motivations, researcher behaviour, personality traits and academic freedom. No survey questions forced a response, and participants had the option to skip any item they were not comfortable answering. Details of item development, piloting, and revisions are provided in the Supplementary Material (Additional File 1, Section S1.1).

### Variables

#### Questionable research practices

I developed a repository of twenty-four QRPs, drawing on previous research examining the prevalence and predictors of QRPs. Item development and source information for QRP items is reported in Additional File 1, Table S1.1. Some items were informed by the broader literature on QRPs and adapted for clarity and relevance across disciplinary and national contexts, while others were developed specifically for this study to capture practices not clearly represented in prior work. Of these, eight practices pertained to ‘qualitative’ research, eight pertained to ‘quantitative’ research and the remaining eight were relevant to all research methodologies. Respondents received a maximum of twelve QRP items and a minimum of eight, determined by their prior indication of methodological approach. Descriptions of these QRPs can be found in Table 1.

**Table 1:** Questionable research practice items from the international survey on research culture and integrity

	**Questionable Research Practice (QRP) question wording**
General QRPs	
QRP 1	Choosing references for citation in a way that primarily supports my own findings or beliefs
QRP 2	Including individuals as authors on a paper who may not have contributed significantly to the research
QRP 3	Citing literature without having read it
QRP 4	Making adjustments to the analysis, inferences or conclusions in a manuscript that you do not believe are valid in order to satisfy reviewers
QRP 5	Omitting citations of publications that present differing views or findings from one’s own work
QRP 6	Using content from papers under review for personal research or publication
QRP 7	Not disclosing relevant conflicts of interest
QRP 8	Excluding junior researchers from the list of authors on a paper despite them having made significant contributions
Quantitative QRPs	
QRP 9	Neglecting to conduct a power analysis to determine the necessary sample size based on the intended significance level, statistical power, and anticipated effect size
QRP 10	Not fully disclosing and making accessible all underlying data, computer codes, or syntaxes used in research on open science platforms
QRP 11	Reporting a set of results as the complete set of analyses when other relevant analyses were also conducted
QRP 12	Presenting an unexpected finding or exploratory analysis in a research report as if it were an a priori hypothesis
QRP 13	Deciding not to report covariates that failed to reach statistical significance (e.g., p < 0.05) or some other desired statistical threshold
QRP 14	Deciding to exclude data points after first checking the impact on statistical significance (e.g., p < 0.05) or some other desired statistical threshold
QRP 15	Rounding off p-values so they appear to be below or equal to the desired alpha (e.g., reporting p = 0.054 as p = 0.05)
QRP 16	Extending data collection or manipulating data selection during an analysis with the specific intent of obtaining statistically significant results where none were previously found
Qualitative QRPs	
QRP 17	Not maintaining comprehensive and detailed documentation of all decisions, modifications, and steps undertaken throughout the research process
QRP 18	Not engaging in reflexivity, which involves failing to acknowledge or address my biases, perspectives, and potential influence on the research process
QRP 19	Failing to sufficiently describe the methods of data collection and analysis, making it difficult for others to understand how conclusions were reached
QRP 20	Phrasing questions in interviews or focus groups in a way that might influence participants towards certain responses
QRP 21	Interpreting qualitative data in a way that confirms the analyst’s pre-existing ideas and beliefs, ignoring data that might suggest alternative interpretations
QRP 22	Deliberately omitting or failing to discuss data that contradict or do not align with the emerging themes or patterns identified in the research
QRP 23	Presenting interview or focus group findings in a manner that may not fully reflect the original data
QRP 24	Providing false or misleading information to participants about the purpose or nature of the study

Respondents were asked to indicate the frequency with which they have carried out the practice over the past three years on a five-point ordinal scale with response alternatives including never, rarely, sometimes, often, always and ‘does not apply in my case’. The three-year timeframe was used to reduce recall bias and ensure prevalence could be stated in more precise terms [10, 11]. The first dependent variable is mean self-reported QRP engagement, with mean score calculated based on all QRPs deemed to apply as indicated by the respondent. Higher scores indicate greater average engagement. This operationalisation is consistent with past research that has modelled the relationship between QRPs and various explanatory factors [14, 20]. Mean self-reported QRP engagement was conceived as a composite indicator of heterogeneous practices rather than as a reflective scale assumed to represent a single latent dimension. The resulting score should therefore be interpreted as an index of average self-reported engagement across the included QRP items, rather than as evidence that all practices form a single latent construct or share identical antecedents. Accordingly, high internal consistency or a simple factor structure was not assumed a priori.

The second dependent variable is a dichotomous indicator of ‘frequent’ QRP engagement, defined as reporting that at least one QRP had been engaged in often or always within the same three-year reference period. This operationalisation was preregistered and is consistent with prior QRP survey research [14]. It was included not as a preferable alternative to the continuous score, but as a complementary indicator capturing whether respondents reported relatively high-frequency engagement in any single QRP. Because both outcomes refer to the same three-year timeframe, they differ in scoring rather than recall period.

The QRP items reflect practices commonly regarded as questionable, but whether a given instance is genuinely problematic may depend on the study design, analytic framework, and the extent to which the decision was prespecified, justified, and transparently reported. This is relevant, for example, where standard reporting conventions may overlap with potentially misleading presentation. Accordingly, the binary QRP measure may classify some contextually defensible cases as QRP engagement and should therefore be interpreted with caution.

#### Publication and funding pressure

I specified a two-factor model (publication vs funding pressure) a priori and tested it using confirmatory factor analysis (CFA). Some of the items measuring publication and funding pressure were adapted from Gopalakrishna et al. [14], while others were newly created for this study. Additional File 1, Table S1.1 reports item development and source information for these items. Based on the confirmed factor-structure, I computed composite scores by reverse coding relevant items and averaging for each respondent. Higher scores indicated greater publication and funding pressure. Mean scores for publication and funding pressure, along with Cronbach’s Alpha values, are reported in Table 2. Full item loadings and fit statistics for publication and funding pressure items are provided in Additional File 1, Table S1.2 and Table S1.3. Mean scores for funding and publication pressure, stratified by field, career stage and sex are reported in Additional File 1, Table S1.4.

**Table 2:** Mean scores (standard deviations) of explanatory factors and scales by disciplinary field, career stage and sex

	**Disciplinary field**				**Career stage**				**Sex**		**Overall**	**Cronbach’s α**	**McDonald’s ω**
**Explanatory Factor Scale**	**Natural sciences**	**Medical sciences**	**Social sciences**	**Humanities**	**Early** **career**	**Mid** **career**	**Late** **career**	**Retired**	**Male**	**Female**			
Publication pressure	4.37 (0.03)	4.17 (0.05)	4.37 (0.03)	4.33 (0.04)	4.64 (0.04)	4.44 (0.03)	4.16 (0.03)	3.82 (0.05)	4.24 (0.02	4.47 (0.03)	4.33 (0.02)	0.70	0.71 (0.00)
Funding pressure	4.84 (0.04)	4.57 (0.06)	4.34 (0.04)	4.22 (0.06)	5.03 (0.05)	4.68 (0.04)	4.23 (0.04)	3.90 (0.07)	4.41 (0.03)	4.70 (0.04)	4.54 (0.02)	0.71	0.72 (0.00)
Universalism	4.27 (0.02)	4.03 (0.03)	3.97 (0.02)	3.91 (0.03)	4.13 (0.03)	4.12 (0.02)	4.06 (0.02)	3.87 (0.04)	4.08 (0.02)	4.09 (0.02)	4.08 (0.01)	0.59	0.61 (0.00)
Communalism	4.06 (0.02)	3.95 (0.03)	3.92 (0.02)	3.96 (0.03)	4.00 (0.03)	3.97 (0.02)	3.99 (0.02)	4.00 (0.04)	4.04 (0.02)	3.90 (0.02)	3.98 (0.01)	0.52	0.54 (0.00)
Organised scepticism	4.67 (0.01)	4.78 (0.02)	4.71 (0.01)	4.76 (0.02)	4.68 (0.02)	4.71 (0.01)	4.74 (0.01)	4.72 (0.02)	4.69 (0.01)	4.76 (0.01)	4.72 (0.01	0.48	0.49 (0.00)
Achievement motivation	3.71 (0.04)	3.83 (0.07)	3.69 (0.04)	3.43 (0.06)	3.92 (0.05)	3.76 (0.04)	3.61 (0.05)	3.02 (0.09)	3.59 (0.03)	3.80 (0.04)	3.67 (0.02)	0.83	0.83 (0.00)
Impact & quality motivation	4.76 (0.03)	4.86 (0.05)	4.67 (0.03)	4.60 (0.05)	4.66 (0.04)	4.77 (0.03)	4.72 (0.03)	4.58 (0.06)	4.61 (0.02)	4.85 (0.03)	4.72 (0.02)	0.52	0.53 (0.00)
Ego & esteem motivation	2.80 (0.04)	2.94 (0.07)	3.05 (0.04)	3.08 (0.06)	2.5 (0.05)	2.95 (0.04)	2.96 (0.04)	2.88 (0.09)	2.85 (0.03)	3.07 (0.04)	2.95 (0.02)	0.58	0.64 (0.00)
Career growth motivation	3.41 (0.04)	3.40 (0.07)	3.52 (0.05)	3.56 (0.06)	3.93 (0.05)	3.60 (0.04)	3.31 (0.05)	2.52 (0.08)	3.34 (0.03)	3.64 (0.04)	3.47 (0.03)	0.74	0.75 (0.00)
Falsification orientation	2.86 (0.05)	3.05 (0.07)	2.66 (0.05)	2.80 (0.07)	2.79 (0.06)	2.76 (0.04)	2.85 (0.05)	2.98 (0.09)	2.91 (0.04)	2.70 (0.04)	2.82 (0.03)	0.83	0.85 (0.00)
Confirmation orientation	3.62 (0.05)	3.70 (0.07)	3.10 (0.05)	3.40 (0.07)	3.50 (0.07)	3.34 (0.05)	3.45 (0.05)	3.67 (0.09)	3.41 (0.04)	3.49 (0.04)	3.44 (0.03)	0.81	0.81 (0.00)
Extraversion	3.98 (0.04)	4.21 (0.07)	4.26 (0.05)	4.22 (0.06)	4.00 (0.06)	4.10 (0.04)	4.28 (0.05)	4.23 (0.08)	4.00 (0.04)	4.33 (0.04)	4.14 (0.03)		
Agreeableness	4.66 (0.04)	4.88 (0.05)	4.82 (0.04)	4.99 (0.05)	4.75 (0.05)	4.78 (0.03)	4.85 (0.04)	4.88 (0.07)	4.66 (0.03)	4.99 (0.03)	4.80 (0.02)		
Conscientiousness	5.68 (0.04)	5.97 (0.05)	5.81 (0.04)	5.95 (0.05)	5.63 (0.05)	5.78 (0.03)	5.93 (0.04)	5.96 (0.06)	5.71 (0.03)	5.95 (0.03)	5.81 (0.02)		
Emotional stability (neuroticism)	5.08 (0.04)	5.47 (0.06)	5.07 (0.04)	5.10 (0.06)	4.85 (0.06)	5.10 (0.04)	5.29 (0.05)	5.41 (0.07)	5.24 (0.03)	5.02 (0.04)	5.14 (0.02)		
Openness	5.72 (0.03)	5.75 (0.05)	5.67 (0.03)	5.92 (0.04)	5.57 (0.04)	5.68 (0.03)	5.88 (0.03)	5.97 (0.05)	5.73 (0.02)	5.77 (0.04)	5.75 (0.02)		

Explanatory factor scales are unstandardised

Publication pressure and funding pressure are composite scales ranging from 1 to 5 with higher scores indicating greater pressure. Universalism, organised scepticism and communalism are composite scales ranging from 1 to 5 with higher scores indicating greater commitment. Personality traits were measured using composite scales ranging from 1 to 6, where higher scores indicate a stronger presence of the trait. Achievement motivation, impact and quality motivation, ego and esteem motivation, career growth motivation, falsification orientation and confirmation orientation are composite scales ranging from 1 to 6 with higher scores indicating greater motivation/orientation

#### Scientific norms

The survey included twenty items designed to measure adherence to Mertonian scientific norms. Each of the four norms – Universalism, Disinterestedness, Organised Scepticism, and Communalism – were measured using five items. These items were developed based on established literature measuring these constructs [23, 30], refined through expert feedback and tested through piloting with a small sample of participants. Respondents were asked to indicate the extent to which twenty items *should* represent the behaviours and views of researchers in the respondent’s field. Respondents indicated an answer on a five-point ordinal scale, with response alternatives ‘never’, ‘rarely’, ‘sometimes’, ‘often’ and ‘always’. I adopted a normative (or prescriptive) measurement strategy, where respondents were asked to evaluate how researchers should ideally behave rather than describing their actual behaviour. This approach captures researchers’ perceptions of the ideal standards of scientific conduct and aligns with past scholarship that has operationalised scientific norms in a similar manner [23]. Details on item development are included in Additional File 1, Table S1.1.

An exploratory factor analysis (EFA) was conducted to examine the underlying structure of these normative constructs. Following item reduction, the final retained solution comprised 10 items loading onto three interpretable factors: universalism, communalism, and organised scepticism. Items intended to capture disinterestedness did not form a distinct retained factor and were therefore not included as a separate composite scale. A three-factor solution was retained based on the factor loadings and theoretical interpretability. Composite reliabilities ranged from α = 0.48—0.59 (see, Additional File 1, Table S1.5). Items loading onto each respective factor were averaged to create composite scores, computing a mean score for each respondent for Communalism, Organised Scepticism, and Universalism. Higher scores indicated greater adherence to the scientific norm. Internal consistency was low for some factors, particularly Organised Scepticism (Cronbach’s α = 0.48) and Communalism (Cronbach’s α = 0.52), indicating limited internal consistency of these item sets. This may reflect measurement limitations, but it may also suggest that these normative constructs are more heterogeneous or less coherently expressed in the present sample than classical formulations would imply. Findings involving these factors should therefore be interpreted with caution. Further psychometric details for the scientific norms are provided in Additional File 1, Table S1.5 (KMO, Bartlett’s test, Cronbach’s alpha, variance explained, and the full pattern matrix).

#### Personal, proximal-scientific motivations and scientific approach

The survey included eighteen items measuring researcher motivations and orientations. There were three broad categories of items, developed through an iterative process, incorporating insights from discussions with researchers, a review of relevant scholarship and reflections on contemporary research practices. The approach ensured that the items captured both theoretical constructs and practical considerations relevant to research motivations and epistemic conditions. These categories included personal motivations, proximal science-specific motivations and approaches to research (i.e., falsification vs. confirmation). Respondents were asked to indicate the extent to which they found each statement (e.g., ‘advancing my career or achieving promotion, compared to other personal and professional motivations’) motivating on a seven-point ordinal scale ranging from 0 (‘Not motivating at all’) to 6 (‘Extremely motivating’). Full question wording and mean scores stratified by disciplinary field, career stage and sex are included in Additional file 1, Table S1.6. Some deviations from the preregistered hypothesis structure reflect the fact that the final composite predictors were derived from empirical scale development and factor analysis, which consolidated or altered some of the originally anticipated motivation constructs.

An exploratory factor analysis was conducted to identify distinct latent constructs pertaining to personal motivations, proximal science-specific motivations and research approaches. The factor analysis yielded two personal motivation factors (career growth; ego & esteem), two proximal science-specific motivation factors (academic/scientific achievement; impact & quality), and two epistemic orientation factors (falsification; confirmation). Composite reliabilities ranged from α = 0.52—0.83. Internal consistency was also low for Ego/Esteem Motivation (Cronbach’s α = 0.58) and Impact & Quality Motivation (Cronbach’s α = 0.52), indicating limited internal consistency of this item set. This may reflect measurement limitations, but it may also suggest that the construct is expressed more heterogeneously in the present sample than a more internally consistent scale would imply. Full psychometric details for the motivation factors are provided in Additional file 1, Table S1.7 (KMO, Bartlett’s test, Cronbach’s alpha, variance explained, and the full pattern matrix).

#### Personality traits

I used the Ten-Item Personality Inventory (TIPI) [31] to measure the Big Five personality traits, including extraversion, agreeableness, conscientiousness, emotional stability (opposite of neuroticism), and openness to experience. The TIPI was chosen for its brevity and suitability for large-scale surveys while effectively capturing the core dimensions of the Big Five personality traits. Despite its short length, the inventory has demonstrated acceptable convergent validity with established Big Five measures [32].

The measure consists of ten items, with two per trait (one positively and one negatively worded), such as *‘*extraverted, enthusiastic’ (for extraversion). Respondents were asked to indicate the extent to which they agreed or disagreed with each descriptor pair on a 7-point Likert scale (1 = Disagree strongly, 7 = Agree strongly). Negatively worded items were reverse-coded, and scores for each trait were computed by averaging responses for the two relevant items.

#### Demographic variables

Given that the sample is derived from those who completed IRIS [16], responses were matched to previously collected demographic data. On this basis, only an updated measure of career stage, and a new measure of methodological approach were collected. Career stage was measured by asking respondents to select from early career, mid-career, late career or retired. This variable was recoded into three dummy variables, with ‘mid-career’ as the reference category. Respondents were asked whether they primarily use a quantitative, qualitative or a mixed-method approach. This variable was recoded into two dummy variables, with quantitative as the reference category. I used demographic measures of sex, discipline and country of employment, as measured in IRIS [16].

In IRIS, each respondent reported their sex, with response categories male, female and prefer not to say. This variable was recoded into a dummy variable, with males as the reference category. Those selecting ‘prefer not to say’ were removed listwise. Disciplinary field was recoded into three dummy variables, with natural sciences as the reference category. Respondents were additionally grouped into ‘United States of America’, ‘Europe’ and ‘other’, based on their self-reported country of employment. This variable was recoded into a dummy variable with ‘Europe’ as the reference category. I additionally asked respondents to indicate where they position themselves on a left–right political scale, ranging between 0–6 (7-point Likert scale), with the label ‘far left’ anchored at 0, ‘far right’ anchored at 6 and ‘centre’ anchored at 3. All continuous composite variables were standardised to z-scores with mean zero and standard deviation one prior to modelling.

#### Survey weights

Survey weights were constructed to adjust for differential selection and nonresponse bias. The use of survey weights was not explicitly specified in the preregistration, but weights were applied to better reflect the intended survey design and reduce potential nonresponse bias. Respondents who indicated willingness to participate were randomly selected with a fixed probability of 0.5, and the likelihood of providing a usable response (defined as completing at least 50% of the survey) was estimated using a logistic regression model that included country, field group, field, and their interactions. Raw weights were derived as the inverse of the product of this fixed selection probability and the predicted response probability, and these weights were subsequently normalised to have a mean of one. This approach ensures that the subsequent analyses accurately reflect the intended survey design while mitigating biases associated with nonresponse.

### Missing data

Data were assumed to be Missing at Random (MAR), therefore Multiple Imputation with mice in R (version 2023.09.0 + 463) was used to produce fifteen imputed datasets [32]. Predictive mean matching was used for continuous variables, proportional odds logistic regression for ordinal variables (including Likert scales) and multinomial logistic regression was used for unordered categorical variables. Models were fit to the fifteen imputed datasets, and the results were combined into a single inference. To account for uncertainty all estimates were combined using Rubin’s rules [33]. Complete case analyses were conducted as a sensitivity check for all models. Missingness in the QRP items had multiple sources. First, respondents were administered different QRP blocks depending on their empirical and methodological profile, creating structural missingness by design. Second, some eligible items were answered with “Does not apply in my case,” which was treated as an observed response rather than missing for diagnostic purposes. Third, some eligible items remained genuinely unobserved. Among empirical quantitative and qualitative respondents, participants provided on average approximately 9.4–9.5 substantive responses, 0.9 “does not apply” responses, and 1.6 genuinely missing responses out of 12 eligible items. Thus, although selective nonresponse on sensitive items remains plausible, unobserved QRP values cannot be interpreted solely as concealment. To reduce instability in the composite outcomes, analyses were restricted to respondents with at least four substantive responses on eligible QRP items. As an additional sensitivity analysis, I repeated the principal substantive analyses using a stricter inclusion criterion, restricting the sample to respondents with at least six substantive responses on eligible QRP items; this reduced the analytic sample only marginally and did not materially change the results.

### Analysis strategy

RStudio was used for all analyses (Version 2025.05.1 + 513). Descriptive statistics of demographic variables were calculated for each of the 15 imputed datasets and pooled by averaging across the imputed datasets. I analysed self-reported QRP engagement (linear regression) and any frequent self-reported QRP engagement (logistic regression) as functions of publication and funding pressure, personal and proximal-scientific motivations, epistemic orientation and the five personality traits, along with my three scientific norm scales and political orientation examined exploratorily. Models 1 A and 1B show coefficient estimates for mean frequency of self-reported engagement in QRPs, while Models 2 A and 2B provide odds ratios for any self-reported frequent QRP engagement. Interaction terms were incorporated into Models 1B and 2B to assess potential moderating effects.

Additional exploratory interaction terms were added to Models 1B and 2B to examine whether publication pressure moderated the associations between self-reported QRP engagement and two sets of individual-level predictors: scientific norms and personality traits. These analyses were distinct from the preregistered moderation hypotheses concerning personal and science-specific motivations and should be interpreted as hypothesis-generating. They were included to explore whether publication pressure conditions the behavioural relevance of individual-level characteristics. For example, scientific norms and traits such as conscientiousness or agreeableness may act as protective factors against QRP engagement, particularly under conditions of heightened publication pressure; alternatively, high publication pressure may weaken these associations if systemic incentives override individual-level dispositions.

Models 3 A and 3B examined whether publication pressure and funding pressure moderate the relationship between the personal motivations (i.e., career growth, and ego and esteem) and proximal science-specific motivations (i.e., achievement, and impact and quality). Here, I additionally include interaction terms for pressure items and a) scientific norms, and b) personality traits for exploratory analyses.

To assess whether proximal-science specific motivations mediate the relationship between personal motivations and QRP engagement, I used structural equation modelling (SEM; lavaan). Personal motivations (PM) of career growth and ego and esteem were included as predictors, while the proximal science-specific motivations of achievement motivation and impact and quality were included as mediators (M). Mean self-reported QRP engagement was included as the outcome. Direct paths from personal motivations to self-reported QRP engagement were retained to allow for the possibility of partial rather than full mediation. The SEM models were estimated to test H12-H19. All variables were treated as manifest, and the models were estimated using maximum likelihood estimation. Model 4B removed non-significant demographic predictors, while the final model (Model 4 C) added a covariance between the two proximal-science specific mediators. Because the substantive conclusions were unchanged across models, only the final model is presented in the main text, with earlier specifications reported in Additional File 1, Section S2.4.

The analyses were organised around the specific theoretical questions under examination rather than a single omnibus model designed to rank predictors by relative importance. A single fully integrated model containing all direct, indirect, and conditional pathways was not estimated because it would combine distinct estimands and would not provide a straightforward test of the preregistered hypotheses. In particular, entering proximal science-specific motivations alongside personal motivations as ordinary covariates would estimate residual direct associations rather than the theoretically specified indirect pathways.

All models include discipline, career-stage, methodological approach, sex and country; applied survey weights; and bootstrapped confidence intervals. The bootstrap was used to estimate within-imputation uncertainty under the survey-weighted design; Rubin’s rules were then used to combine estimates and standard errors across imputations. Full analysis code is available on OSF [28].

## Results

### Descriptive statistics

A small majority of the respondents were male (57%), 37.2% primarily research in the natural sciences, 14.0% in the medical sciences, 31.8% in the social sciences and 18.0% in the humanities and arts. 19.7% were early-career, 41.1% were mid-career, 29% were late career and 10.2% were retired. Moreover, 4.4% were based in the United States, 84.2% were based in Europe and 11.4% were based elsewhere.

**Fig. 3:**
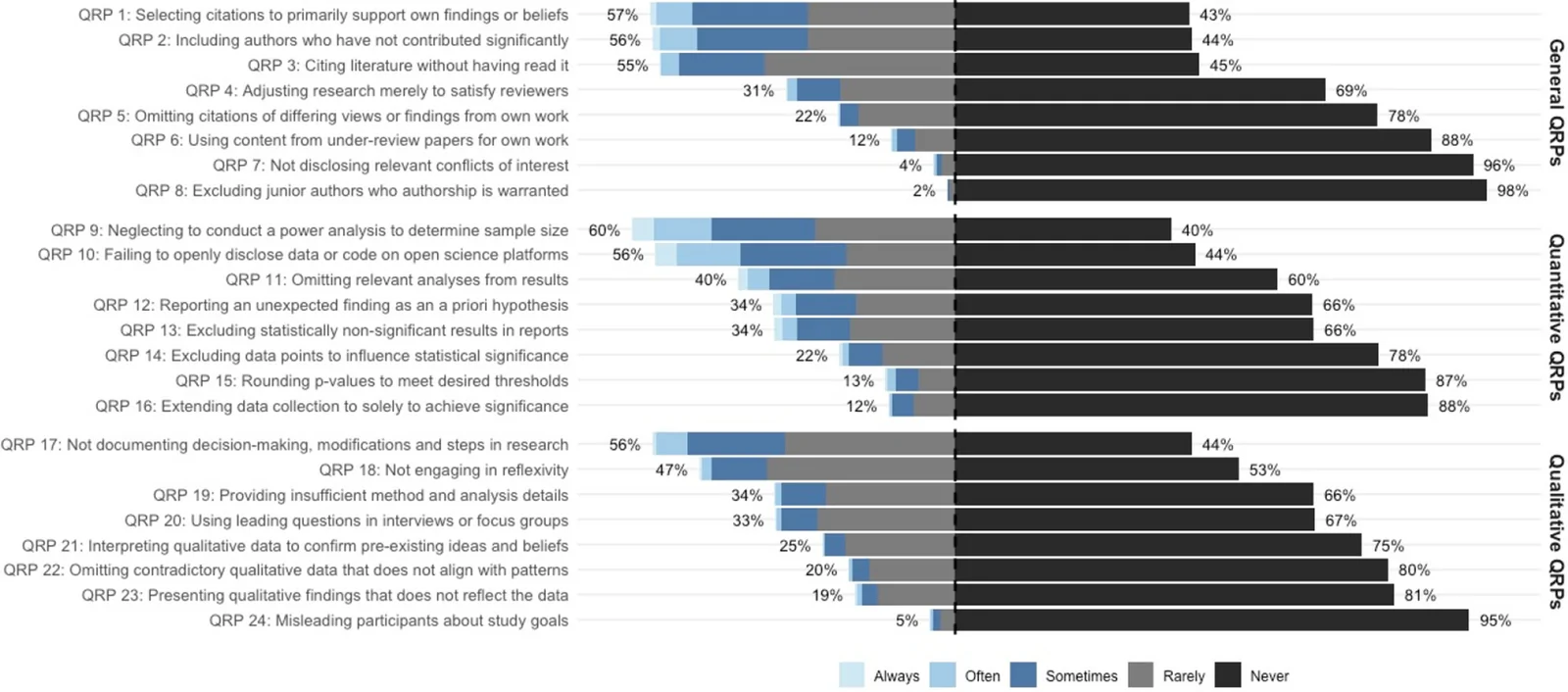
Percentage of observed answer categories of QRPs across 3088 respondents

Figure 3 shows the percentage of observed answer categories for the individual self-reported QRP items, grouped by quantitative, qualitative and general methodological approaches, while percentages for each answer category and confidence intervals for all QRP items are shown in Additional File 1, Table S2.1.

Descriptive statistics for unstandardised dependent variables are shown in Table 3. Mean self-reported QRP engagement averaged at the lower end of the scale, and showed limited variation by field, career stage and sex. The proportion reporting frequent engagement in at least one QRP was broadly similar across disciplinary fields, though notably lower in the humanities. There was clearer variability in any frequent self-reported QRP engagement across career stages, with lower levels among late-career individuals and the retired. Descriptive statistics for explanatory scales are included in Table 2. Across these subgroups, both publication and funding pressure averaged at the higher end of the scale, indicating that researchers perceived substantial systemic pressures. Endorsement of universalism, communalism and organised scepticism were high across all demographics. Among motivations, impact and quality was the most strongly endorsed on average. Bivariate correlations among the study variables are reported in Additional File 1, Table S2.2.

**Table 3:** Mean QRP (SD) and any frequent QRP by disciplinary field, career stage and sex

	**Disciplinary field**				**Career stage**				**Sex**		**Overall**
**Explanatory factor scale**	**Natural sciences**	**Medical sciences**	**Social sciences**	**Humanities**	**Early career**	**Mid career**	**Late career**	**Retired**	**Male**	**Female**	
**Mean QRP**	1.49 (0.36)	1.49 (0.39)	1.5 (0.37)	1.41 (0.35)	1.57 (0.43)	1.49 (0.37)	1.41 (0.33)	1.42 (0.36)	1.49 (0.37)	1.47 (0.38)	1.48 (0.37)
**Any frequent QRP** (% Having Engaged)	30.54	32.24	31.60	17.02	36.94	31.12	22.23	21.83	28.77	28.31	28.73

*n* = 3050; Mean QRP is unstandardised and computed based on 4–12 QRP self-report items with not applicable responses removed; Unstandardised Mean QRP is measured on a 1–5 scale, with higher scores indicating greater engagement; Any frequent QRP is at least one QRPs being often or always being engaged in

### Regression analyses

Table 4 shows the multivariate regression estimate for the five demographic characteristics. The multivariate regression results for Models 1 A, 1B, 2 A and 2B are shown in Table 5. Models 1 A and 1B show coefficient estimates for mean frequency of self-reported engagement in QRPs, while Models 2 A and 2B provide odds ratios for any self-reported frequent QRP engagement. Interaction terms were incorporated into Models 1B and 2B to assess potential moderating effects. All models contained the five background characteristics.

**Table 4:** Regression Coefficients (95% CI) of mean QRP and frequent QRP by demographic characteristics

	**Overall mean QRP**	**Any frequent QRP**
	**Linear regression model** **Coefficient (95% CI)**	**Logistic regression model** **OR (95% CI)**
Disciplinary field (reference: natural sciences)		
Medical sciences Social sciences Humanities	0.03 (−0.08, 0.14) −0.02 (−0.11, 0.07) −0.18 (−0.29, −0.08)***	1.15 (0.88, 1.50) 1.09 (0.87, 1.36) 0.61 (0.45, 0.82)**
Career stage (reference: mid-career)		
Early career Late career Retired	0.12 (0.02, 0.22)* −0.14 (−0.22, −0.06)*** −0.09 (−0.22, 0.03)	1.19 (0.95, 1.47) 0.68 (0.55, 0.85)*** 0.73 (0.53, 1.02)
Methodological approach (reference: quantitative)		
Qualitative Mixed-methods	−0.20 (−0.32, −0.08)** −0.12 (−0.20, −0.04)**	0.48 (0.35, 0.64)*** 0.73 (0.60, 0.89)**
Sex (reference: male)		
Female	−0.04 (−0.12, 0.03)	0.95 (0.79, 1.14)
Country (reference: Europe)		
United States Other	−0.01 (−0.16, 0.14) −0.05 (−0.17, 0.07)	1.33 (0.87, 2.03) 0.98 (0.74, 1.30)

*n* = 3050; Estimates from Models 1B and 2B, including all independent variables and interactions. Coefficients and confidence intervals (CIs) are based on pooled estimates across 15 imputed datasets, using Rubin’s rules. CIs are bootstrapped. Sampling weights were included in all models

Overall mean QRP was computed based on 4–12 QRP items with not applicable responses removed

Any frequent QRP is computed based on at least one QRPs being ‘often’ or ‘always’ engaged in

^*^p < 0.05; ** *p* < 0.01; *** *p* < 0.001

**Table 5:** Regression Coefficients (95% CI) of mean QRP and any frequent QRP by explanatory variables

	**Overall mean QRP**		**Any frequent QRP**	
	**Linear regression** **Model 1A** **Coefficient (95% CI)**	**Linear regression** **Model 1B** **Coefficient (95% CI)**	**Logistic regression** **Model 2A** **OR (95% CI)**	**Logistic regression** **Model 2B** **OR (95% CI)**
Publication pressure	0.12 (0.08, 0.15)***	0.12 (0.08, 0.16)***	1.24 (1.12, 1.37)***	1.24 (1.12, 1.38)***
Funding pressure	−0.01 (−0.05, 0.03)	−0.01 (−0.05, 0.03)	1.05 (0.94, 1.16)	1.05 (0.94, 1.17)
Scientific norms				
Universalism Communalism Organised scepticism	−0.15 (−0.19, −0.11)*** −0.04 (−0.08, 0.00) −0.15 (−0.2, −0.1)***	−0.14 (−0.18, −0.1)*** −0.04 (−0.08, 0)* −0.15 (−0.2, −0.1)***	0.9 (0.82, 0.99)* 0.93 (0.85, 1.02) 0.91 (0.83, 0.99)*	0.91 (0.83, 1) 0.93 (0.84, 1.02) 0.90 (0.82, 0.98)*
Personal motivations				
Career growth Ego and esteem	−0.03 (−0.07, 0.02) 0.11 (0.07, 0.15)***	−0.03 (−0.07, 0.01) 0.11 (0.07, 0.15)***	0.9 (0.81, 1.01) 1.09 (0.98, 1.20)	0.9 (0.8, 1.01) 1.08 (0.98, 1.2)
Proximal science-specific motivations				
Impact and quality Academic achievement	−0.1 (−0.15, −0.06)*** 0.06 (0.02, 0.11)***	−0.11 (−0.15, −0.06)*** 0.07 (0.02, 0.11)**	0.92 (0.83, 1.02) 1.12 (0.99, 1.27)	0.92 (0.83, 1.03) 1.13 (1.00, 1.28)
Research approach				
Falsification Confirmation	−0.06 (−0.11, −0.01)* 0.04 (−0.01, 0.09)	−0.06 (−0.11, −0.01)* 0.04 (−0.01, 0.09)	0.85 (0.75, 0.96)** 1.03 (0.91, 1.18)	0.85 (0.75, 0.96)** 1.04 (0.91, 1.18)
Personality traits				
Extraversion Agreeableness Conscientiousness Emotional stability (neuroticism) Openness	0.03 (−0.01, 0.06) −0.04 (−0.07, 0.00)* −0.07 (−0.11, −0.03)*** −0.08 (−0.11, −0.04)*** −0.08 (−0.12, −0.04)***	0.03 (−0.01, 0.06) −0.03 (−0.07, 0.00)* −0.07 (−0.11, −0.03)*** −0.07 (−0.11, −0.04)*** −0.08 (−0.12, −0.04)***	1.03 (0.94, 1.12) 0.9 (0.82, 0.98)* 1.05 (0.96, 1.15) 0.90 (0.82, 0.98)* 0.95 (0.86, 1.04)	1.02 (0.94, 1.13) 0.89 (0.81, 0.98)* 1.05 (0.96, 1.16) 0.92 (0.83, 1.01) 0.94 (0.86, 1.04)
Political orientation	−0.03 (−0.07, 0.02)	−0.03 (−0.07, 0.01)	0.99 (0.91, 1.1)	1.00 (0.91, 1.10)
Universalism * funding pressure		0.02 (−0.02, 0.05)		0.92 (0.84, 1.02)
Universalism * publication pressure		−0.04 (−0.08, 0.00)*		0.96 (0.87, 1.06)
Organised scepticism * funding pressure		0.01 (−0.05, 0.06)		1.00 (0.89, 1.11)
Organised scepticism * publication pressure		−0.02 (−0.02, 0.06)		1.04 (0.95, 1.14)
Communalism * publication pressure		0.01 (−0.03, 0.05)		1.01 (0.92, 1.11)
Communalism * funding pressure		0.01 (−0.03, 0.05)		1.04 (0.95, 1.15)
Conscientiousness * publication pressure		0.03 (−0.01, 0.07)		1.02 (0.93, 1.11)
Emotional stability * publication pressure		−0.03 (−0.08, 0.01)		0.91 (0.83, 1.00)*
Openness * publication pressure		0 (−0.03, 0.04)		1.00 (0.92, 1.09)
Agreeableness * publication pressure		−0.01 (−0.04, 0.02)		1.02 (0.93, 1.12)

*n* = 3050; Coefficients and confidence intervals (CIs) are based on pooled estimates across 15 imputed datasets, using Rubin’s rules. CIs are bootstrapped. Sampling weights were included in all models

Overall mean QRP was computed based on 4–12 QRP items with not applicable responses removed

Any frequent QRP is at least one of the QRPs being often or always being engaged in

All models contain the five background characteristics of sex, disciplinary field, methodological approach, career stage and country

^*^p < 0.05; ** *p* < 0.01; *** *p* < 0.001

Table 4 shows that researchers in the humanities reported engaging in fewer QRPs on average, and were less likely to report frequent QRP engagement, compared to their counterparts in the natural sciences. Average self-reported QRP engagement decreased as researchers move through their careers, with those earlier in their career reporting greater average QRP engagement compared to researchers in the middle of their career. Self-reported QRP engagement averaged lower among qualitative and mixed-method researchers compared to quantitative researchers, while quantitative researchers were also more likely to report having engaged in a QRP frequently compared to their qualitative and mix-methods counterparts. I found no statistically significant sex or country differences.

Table 5 shows that one standard deviation increase in publication pressure was associated with a 0.12 (*p* < 0.001) standard deviation increase in average self-reported QRP engagement. Likewise, I found that for every standard deviation increase in publication pressure, the odds of reporting to have engaged in at least one QRP frequently increased by 1.24 (*p* < 0.001). Contrary to H10, I found no statistically significant association between funding pressure and a) self-reported average QRP engagement and b) any self-reported frequent QRP engagement. A greater commitment to universalism, communalism and organised scepticism was associated with less average self-reported QRP engagement. Universalism and organised scepticism were the strongest correlates of self-reported QRP engagement across all models, with a one standard deviation increase in commitment associated with a 0.14 and 0.15 (*p* < 0.001) standard deviation decrease in average self-reported QRP engagement, respectively. Greater commitment to organised scepticism was additionally associated with 0.90 (*p* = 0.02) decreased odds of reporting to have engaged in at least one QRP frequently. There was a statistically significant interaction between publication pressure and universalism in predicting self-reported QRP engagement (β = –0.04, *p* = 0.049). The negative coefficient indicates that the association between publication pressure and average self-reported QRP engagement weakens as commitment to the normative ideal of universalism increases. That is, commitment to universalism appeared to modestly attenuate the association between publication pressure and self-reported QRP engagement; however, the effect size is very small.

A standard deviation increase in ego and esteem motivation was associated with a 0.11 (*p* < 0.001) standard deviation increase in average self-reported QRP engagement. Moreover, where a researcher is more motivated by impact and quality, average self-reported QRP engagement was lower, with a 0.11 (*p* < 0.001) standard deviation decrease in average self-reported QRP engagement for a one standard deviation increase on the impact and quality motivation scale. Conversely, where a researcher was more motivated by academic achievement (i.e., higher citation indices, more frequent publication) average self-reported QRP engagement was greater, with a 0.07 (*p* = 0.007) standard deviation increase in average self-reported QRP engagement for each standard deviation increase on the achievement motivation scale. A researcher primarily orientated towards 'falsifying' theories and findings was associated with a decreased average self-reported QRP engagement (β = –0.06, *p* < 0.001). Moreover, for each standard deviation increase on the falsification scale the odds of reporting to having engaged in a QRP frequently decreases by a factor of 0.85 (*p* < 0.001). I found no statistically significant relationship between a confirmatory research approach and self-reported QRP engagement. Higher levels of agreeableness, conscientiousness, emotional stability (opposite to neuroticism) and openness were associated with lower average self-reported QRP engagement. While I found no statistically significant direct relationship between neuroticism and any frequent QRP engagement, the interaction between neuroticism and publication pressure was significant (OR = 0.91, p = 0.02). This indicated that neuroticism to some extent moderates the relationship between publication pressure and frequent self-reported QRP engagement. Given that higher scores reflect greater emotional stability, the positive effect of publication pressure on frequent self-reported QRP engagement was stronger among researchers who are less emotionally stable.

### Moderation analyses

Table 6 shows the results of the regression analyses for Models 3 A and 3B. I found no statistically significant moderating role of publication pressure nor funding pressure on the relationship between the personal motivations and proximal science-specific motivations. However, I found a statistically significant association between publication pressure, funding pressure and the personal motivations and both the proximal-science specific motivations of achievement and impact/quality.

**Table 6:** Regression Coefficients (95% CI) of proximal science-specific motivators (achievement motivation and impact and quality motivation)

	**Achievement Motivation**	**Impact and Quality Motivation**
	**Linear Regression** **Model 3A** **Coefficient (95% CI)**	**Linear Regression** **Model 3B** **Coefficient (95% CI)**
**Publication pressure**	0.08 (0.04, 0.12)***	0.01 (−0.03, 0.05)
**Funding pressure**	0.14 (0.10, 0.18)***	0.05 (0.00, 0.09)*
**Personal motivations**		
Career Growth Ego and Esteem	0.34 (0.30, 0.37)*** 0.25 (0.22, 0.29)***	0.05 (−0.00, 0.09) 0.15 (0.11, 0.19)***
Career growth * funding pressure	−0.02 (−0.06, 0.01)	0.00 (−0.04, 0.05)
Career growth * publication pressure	0.02 (−0.01, 0.06)	0.00 (−0.04, 0.05)
Ego and esteem * funding pressure	0.00 (−0.04, 0.03)	0.00 (−0.04, 0.05)
Ego and esteem * publication pressure	−0.03 (−0.07, 0.01)	−0.04 (−0.09, 0.01)

*n* = 3050; Coefficients and confidence intervals (CIs) are based on pooled estimates across 15 imputed datasets, using Rubin’s rules. CIs are bootstrapped. Sampling weights were included in all models. Models include five background characteristics (sex, disciplinary field, methodological approach, career stage and country) and left–right political orientation scale

^*^ *p* < 0.05; ** *p* < 0.01; *** *p* < 0.001

### Mediation analyses

Tables 7 and 8 show the results from the mediation analysis, which is the structural equation models examining the theorised relationships among personal motivations and proximal science-specific motivations, and average self-reported QRP engagement. Path coefficients and fit indices for all models, including development models, are contained in Additional File 1, Section S2.4. Specifically, Table 7 shows direct effects and fit statistics, and Table 8 shows the indirect effects.

**Table 7:** Path coefficients (95% CIs) and fit statistics for structural model of direct effects

	**Standardised coefficient (95% CI)**
Career growth motivation (X1) → achievement motivation (M1)	0.39 (0.36, 0.43) ***
Ego and esteem motivation (X2) → achievement motivation (M1)	0.26 (0.23, 0.30) ***
Career growth motivation (X1) → Impact and quality motivation (M2)	0.07 (0.02, 0.10)**
Ego and esteem motivation (X2) → Impact and quality motivation (M2)	0.15 (0.11, 0.19) ***
Achievement motivation (M1) → mean QRP (Y)	0.10 (0.06, 0.13) ***
Impact and quality motivation (M2) → mean QRP (Y)	−0.19 (−0.23, −0.15) ***
Career growth motivation (X1) → mean QRP (Y)	
Ego and esteem motivation (X2) → mean QRP (Y)	0.14 (0.10, 0.18) ***
R^2^ Mean QRP	0.09 (0.00)
R^2^ Achievement motivation	0.35 (0.00)
R^2^ Impact and quality motivation	0.08 (0.00)
Tucker-Lewis Index (TLI)	0.98
Root Mean Square Error of Approximation (RMSEA)	0.02
CFI (Comparative Fit Index)	0.99

*n* = 3050; Coefficients and SEs are based on pooled estimates across 15 imputed datasets, using Rubin’s rules

Sampling weights were included in all models

Non-significant demographic controls (*p* >.05) were removed and covariance between M1 and M2 was added, based on theoretical rationale and modification indices

^*^p < 0.05; ** *p* < 0.01; *** *p* < 0.001

**Table 8:** Path coefficients (95% CIs) and fit statistics for structural model of indirect effects

	**Standardised Coefficient (95% CI)**
Career growth motivation (X1) → achievement motivation (M1) → mean QRP (Y)	0.04 (0.02, 0.05) ***
Ego and esteem motivation (X2) → achievement motivation (M1) → mean QRP (Y)	0.03 (0.01, 0.04) ***
Career growth motivation (X1) → Impact and quality motivation (M2) → mean QRP (Y)	−0.01 (−0.02, −0.00)**
Ego and esteem motivation (X2) → Impact and quality motivation (M2) → mean QRP (Y)	−0.03 (−0.04, −0.02)***

*n* = 3050; Coefficients and SEs are based on pooled estimates across 15 imputed datasets, using Rubin’s rules

Sampling weights were included in all models

Non-significant demographic controls (*p* >.05) were removed and covariance between M1 and M2 was added, based on theoretical rationale and modification indices

^*^p < 0.05; ** *p* < 0.01; *** *p* < 0.001

The final structural model yielded an excellent fit to the data (TLI = 0.98, RMSEA = 0.02, CFI = 0.99). The pattern of coefficients was consistent with partial rather than full mediation, as some personal motivations retained direct associations with self-reported QRP engagement alongside statistically significant indirect associations through the proximal science-specific motivations. Results indicated that ego and esteem motivation was positively associated with average self-reported QRP engagement (β = 0.14, p < 0.001), as was the proximal science-specific motivation of scientific achievement (β = 0.10, p < 0.001). By contrast, impact and quality motivation was negatively associated with average self-reported QRP engagement (β = −0.19, p < 0.001). No statistically significant direct effect was observed for career growth motivation, but statistically significant indirect effects were observed through both achievement motivation (β = 0.04, p < 0.001) and impact and quality motivation (β = −0.01, p = 0.005). Ego and esteem motivation likewise showed statistically significant indirect effects through achievement motivation (β = 0.03, p < 0.001) and impact and quality motivation (β = −0.03, p < 0.001), while retaining a positive direct association with self-reported QRP engagement. This pattern suggests that the included mediators capture only part of the relationship between personal motivations and QRP engagement.

### Summary of statistically significant associations

**Fig. 4:**
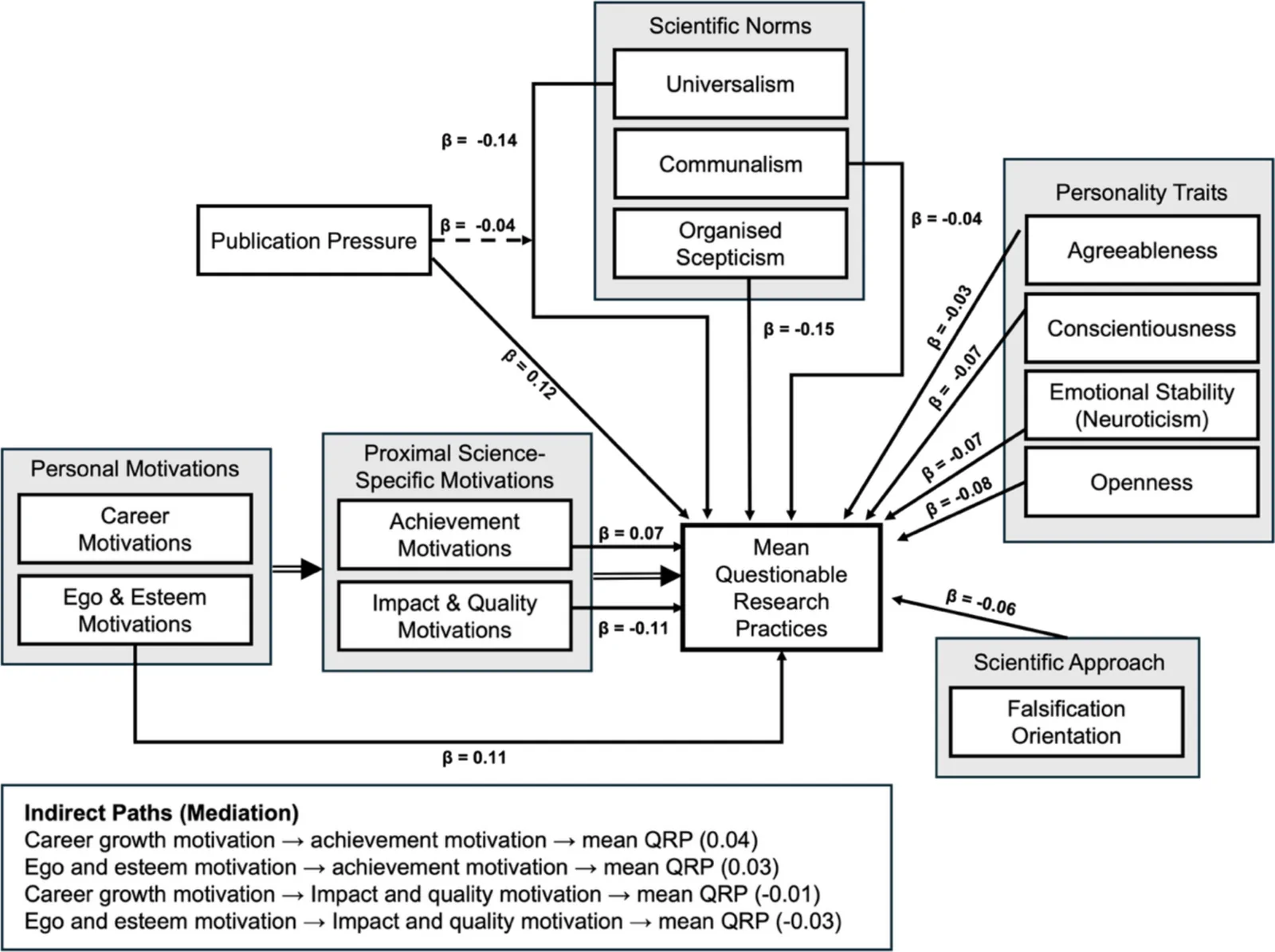
Visual summary of statistically significant associations identified across analyses. Note. The coefficients shown are synthesised from separate analyses rather than derived from a single integrated model. Direct comparisons of effect sizes should be interpreted with caution. Only statistically significant predictors are displayed in this final model. Non-significant paths from the initial conceptual model were removed to yield a parsimonious representation. The compound (double-lined) arrow indicates the indirect paths from personal motivations through proximal science-specific motivations

Figure 4 provides a visual summary of statistically significant associations identified across the separate analyses. It is intended as a descriptive synthesis of the reported results, not as a jointly estimated model or as evidence of the relative independent contribution of each predictor. Only statistically significant predictors are displayed to provide a parsimonious overview of the associations observed in the present study.

## Discussion

In this study, I examined theory-informed associations between motivational, normative, individual-difference, and systemic factors and average self-reported engagement in questionable research practices (QRPs). This research supports pre-eminent theorising that misaligned incentive structures and the resulting systemic pressures, *at least in part*, motivate engagement in QRPs [18]. This aligns with findings from comparable surveys – often conducted within specific disciplinary fields or national contexts – which consistently identify publication pressure as among the strongest correlates of self-reported QRP engagement [14, 15, 34–36]. The observed effect sizes are modest (as is typical in the extant literature) but may reflect restricted variability in self-reported publication pressure in the sample, which would attenuate associations. Moreover, modest individual-level effects can still be meaningful in aggregate when the relevant pressures are widespread and influence repeated research decisions across many researchers and outputs. At the same time, modest associations are also consistent with the possibility that publication pressure is *not* the singular – or even dominant – proximal driver of QRPs in many settings.

I found no evidence to support a significant association between funding pressure and extent of self-reported engagement in QRPs. One possible explanation is that the behaviours captured under the QRP construct – though broad and extensive – may not directly reflect practices that offer a competitive advantage in securing research funding. As such, the link between funding-related pressures and self-reported QRP engagement may be more nuanced or operate through alternative mechanisms not captured in this research.

I also found lower self-reported QRP engagement among late-career and retired researchers. This may reflect reduced exposure to career-related pressures and incentives, but cohort differences in research culture or reporting tendencies may also contribute. Although the cross-sectional design does not allow these explanations to be disentangled, the pattern is consistent with the broader argument that QRP engagement is shaped in part by the incentive structures researchers face.

Scientific norms emerged as one of the strongest correlates of QRP engagement. The greater a researcher's commitment to universalism, communalism and organised scepticism, the less likely they were to report engaging in QRPs, with comparably stronger effects for universalism and organised scepticism than for communalism. These associations were comparable to, and in some cases larger than, the associations observed with publication pressure. The findings here align with prior research demonstrating that stronger commitment to scientific norms – when conceptualised as a unified construct – are associated with lower levels of self-reported engagement in QRPs and scientific misconduct [14, 23, 28]. However, I extend these findings by suggesting that the qualitative characteristics of individual scientific norms matter, with certain normative ideals potentially exerting a stronger influence on QRP engagement than others.

On this basis, I echo recommendations that I have made elsewhere [20] that institutional leaders should perhaps place greater emphasis on fostering commitment to scientific norms as a means of enhancing adherence and promoting high standards of research practice [37, 38]. Again, while the observed associations are modest in magnitude, even small differences *may* be consequential when scaled across large research communities and repeated decisions over time.

I additionally find that epistemic orientation is potentially important in shaping research practice. Researchers who were motivated to falsify their own findings and theories – or those of other researchers – were significantly less likely to report to having engaged in QRPs. This aligns with longstanding scientific ideals, particularly those rooted in Popperian falsification, which emphasise critical scrutiny and the systematic testing of competing explanations. This complements the finding that researchers with a greater commitment to the Mertonian norm of organised scepticism report to having engaged in fewer QRPs on average.

Beyond a researchers 'value-system' and epistemic orientation, I find that their personal and proximal-science specific motivations correlated with self-reported engagement in QRPs. This pattern is broadly consistent with the social-psychological model of researcher practice developed by Jussim and colleagues [27]. The findings reveal an indirect association of heightened career motivation – such as aspirations for increased salary or promotion – with self-reported QRP engagement through a stronger drive for scientific achievement, operationalised here through frequent publication and citation accumulation. These results suggest that career motivation is not inherently problematic; rather, it potentially becomes a risk factor when academic success is defined by metrics that are susceptible to gaming or manipulation. Promoting career advancement frameworks that reward quality-enhancing practices – such as open science, transparency, and methodological integrity – rather than quantity-based output metrics, may offer a viable strategy for reducing engagement in QRPs.

However, an additional indirect pathway points in the opposite direction: career growth ambitions were associated with a stronger motivation to conduct impactful and high-quality research, and this motivation was negatively associated with QRP engagement. This increased focus on impact and quality is negatively associated with QRP engagement, suggesting that when researchers perceive career advancement as tied to the broader scientific or societal impact of their work, they are less likely to compromise methodological integrity. Although this positive pathway is weaker than the publication-metric-driven pathway, it indicates that institutions that effectively align career advancement with research quality and impact may not only discourage QRPs but actively promote a healthier research culture. This is consistent with Janke et al.’s (2019) [39] observation that achievement goals oriented toward skill-development are associated with reduced QRP engagement, while those oriented towards skill demonstration are associated with increased QRP engagement. At the same time, these indirect pathways should be interpreted as partial rather than exhaustive. In particular, ego and esteem motivation retained a direct association with self-reported QRP engagement while also operating indirectly through proximal science-specific motivations, suggesting that the included mediators capture only part of the relationship.

Where researchers were motivated to obtain fame, avoid external perceptions of failure and achieve political or social goals (here termed 'ego and esteem motivations'), average self-reported QRP engagement was higher. These motivations reflect a desire for self-enhancement and external validation which come at cost of epistemic and integrity concerns. On this basis, it is not unreasonable to suggest that research cultures should be motivated by intrinsic, epistemic concerns, rather than extrinsic, image-oriented concerns. Interestingly, we did not find that political orientation influences QRP engagement. On this basis, it’s feasible that a researcher’s political orientation (i.e., whether they’re left- or right- leaning) per se does not matter, but rather the instrumentalisation of research for ideological-related ends feasibly *does* matter. When scientific research becomes a means to advance personal, social or political objectives, the risk of compromised integrity appears to increase.

The proposed moderating role of systemic pressures in the relationship between personal motivations and science-specific motivations was not supported. That is, the results do not support the idea that publication and funding pressure strengthen the translation of broad personal motivations into proximal science-specific motivations. One interpretation is that systemic pressures and individual motivations may operate more additively than interactively. Researchers who experience greater publication or funding pressure may report stronger science-specific motivations regardless of their broader personal motivations, while researchers high in career achievement or ego/esteem motivation may report stronger proximal science-specific motivations regardless of perceived pressure. This pattern is more consistent with independent motivational and systemic pathways than with a conditional amplification process. A second possibility is that publication and funding pressures are sufficiently widespread in contemporary academic environments that there is limited variation in perceived pressure capable of moderating these motivational relationships. Either way, the absence of the preregistered interaction effects suggests that the original moderation component of the theoretical model should be treated cautiously and that the proposed pressure-as-amplifier mechanism was not supported in the present data.

Finally, higher levels of conscientiousness, agreeableness, emotional stability (opposite to neuroticism) and openness among researchers were negatively associated with self-reported QRP engagement. This suggests that researchers who are more diligent, cooperative, emotionally sensitive and open to new experiences are perhaps less inclined to compromise their research integrity and engage in QRPs. This is consistent with earlier research showing that greater conscientiousness, agreeableness, emotional stability and openness may act as protective traits against challenges to research integrity, such as QRPs [15, 21]. Schneider et al. (2024) [15] found only minuscule associations between extraversion and QRP engagement. Similarly, I failed to find a statistically significant relationship between extraversion and self-reported QRP engagement.

### Limitations

This sample comprised individuals who previously participated in IRIS and self-selected as willing to be contacted for future research. As a result, certain characteristics may be overrepresented in the data, potentially limiting representativeness. In addition, because QRP engagement is self-reported and socially sensitive, absolute prevalence estimates may be biased downwards due to underreporting. Conversely, prior IRIS participation may increase respondents’ awareness of what constitutes a QRP and their willingness or ability to recognise and report it, which could inflate estimates relative to a less integrity-aware sample. While these issues bear most directly on prevalence, they may also affect associations. Non-differential misreporting would mainly attenuate associations, whereas differential misreporting correlated with key predictors could bias estimates in either direction. This concern is not limited to any single predictor family. If willingness to disclose QRP engagement varies systematically with motivations, scientific norms, perceived pressures, epistemic orientation, personality traits, or other respondent characteristics, then the observed associations may partly reflect differences in disclosure tendency rather than differences in actual behaviour. This is especially relevant because the study did not include a social desirability measure or external behavioural validation of QRP engagement, meaning that the extent of this potential bias cannot be estimated in the present data. The findings should therefore be interpreted as associations with self-reported QRP engagement specifically. Nonetheless, confidence in the substantive patterns is strengthened by their consistency, in direction and magnitude, with findings in the extant literature, and by the inclusion of relevant controls, including methodological orientation, in the models.

Moreover, given that the sample was drawn predominantly from researchers in the US and Europe, the findings should not be interpreted as globally representative estimates of QRP prevalence. This is particularly important because prevalence estimates vary across surveys and depend substantially on the definitions, thresholds, and sampling frames used. Although the overall response rate was low, it is broadly comparable to similar surveys in this area [10, 14, 40].

A further limitation is that the scientific norm scales showed low internal consistency. As previously discussed, although this may reflect the heterogeneous expression of these norms in the present sample rather than simple measurement failure, it nevertheless suggests that associations involving these constructs may be estimated with greater error and should therefore be interpreted cautiously.

A related limitation concerns the aggregation of heterogeneous QRPs into a single mean outcome. The composite score was used to estimate broad correlates of average self-reported QRP engagement across the included behaviours, not to imply that all QRPs form a unidimensional latent scale or have identical antecedents. It is plausible that specific QRPs are associated with different predictors; for example, publication pressure, methodological approach, career stage, or motivations may matter more for some practices than others. Aggregating across practices may therefore obscure item-specific patterns. The findings should consequently be interpreted as associations with average self-reported QRP engagement, rather than as evidence that the same predictors apply uniformly to each specific QRP.

Effect sizes across predictors are uniformly small. While this is consistent with patterns observed in the extant literature, where correlates of QRP engagement tend to be modest but potentially meaningful, the modest magnitudes reinforce the need for humility in interpretation, particularly considering possible attenuation and nonresponse-related biases.

## Conclusion

The prevalence and downstream impact of QRPs underscore the need for a robust explanatory framework of QRP engagement. Here, I identify a set of individual-level social and psychological predictors that meaningfully explain variation in engagement in these practices. Foremost, the findings suggest that individual-level factors, such as researchers’ normative commitments and personal and scientific motivations, are important alongside systemic-level pressures such as publication pressure in explaining variation in self-reported QRP engagement. I show that publication pressure is an important correlate of QRP engagement, but the findings suggest that it does not independently account for the observed pattern of individual differences. It should therefore be interpreted as one salient correlate within a broader causal ecology rather than as a complete explanation. Mitigating the use of QRPs and maximising the validity of research feasibly requires a multifaceted approach that targets both systemic structures (i.e., ‘publish or perish’ culture) and the individual motivations, values, and beliefs that drive research conduct. A multifaceted, evidence-based approach will permit the mitigation of QRP engagement, and therefore improve the quality, validity and reliability of scientific research.

## Supplementary Information

Additional file 1. 

## Data Availability

The dataset used in this study is not yet publicly available because the author is currently preparing a cleaned, anonymised version suitable for public deposition. The dataset will be deposited in a public data repository upon publication and will be made available on reasonable request in the interim. Analysis code is available on the Open Science Framework.

## Footnotes

1 Despite the variable and contested estimates even a non-zero prevalence rate is a cause for concern, given that they may contribute to the accumulation of biased or fragile scientific findings, and in some cases, plausibly lead to the dissemination of inaccurate media representations, failure of policies and interventions built on the evidence base, and diminished public confidence in science.

2 Merton [22] proposed that scientists adhere to a set of informal cultural norms that guide behaviour, including universalism (i.e., evaluating claims based on objective criteria rather than personal attributes), disinterestedness (pursuing research for the advancement of knowledge rather than personal gain), organised scepticism (subjecting evidence to critical scrutiny), and communalism (promoting the collective ownership and dissemination of scientific knowledge).
