# Supplementary material for: Why researchers cut corners: motivations, pressures, and personality in questionable research practices
Source: Res Integr Peer Rev. 2026 Jul 17;11:58. doi: 10.1186/s41073-026-00229-8 (PMC13670749; doi:10.1186/s41073-026-00229-8)
Supplement: Supplementary file 1 [file 41073_2026_229_MOESM1_ESM.pdf]

<u>**Additional File 1 – Supplementary Material**</u>

S1. Supplementary Methods

S1.1. Instrument Development (Expanded)

Survey questions were meticulously developed between April and November 2024 and the survey was deployed online in November 2024 using Qualtrics – the web-based platform for survey design and administration. All researchers had previously indicated that they hold at least a master's level degree and produce research for commercial or academic organisations within the European Union, European Free Trade Area, United Kingdom or United States. The survey was conducted entirely in English and was initially piloted with twenty-five researchers in October and November 2024. Following piloting, the survey was refined by adjusting several questions and removing items to enhance clarity, focus and reduce completion time. Between November 19th and 20th 2024, a pre-notification email was sent to all individuals in the sample to inform them about the upcoming survey. Invitations to participate were sent between November 21st and 22nd. The invitation emails included information on the project, a link to the survey, an opt-out link removing recipients from further communication, and information on identity protection measures. Upon clicking the survey link, participants were greeted with the participation information sheet, followed by an informed consent page. To encourage completion, two email reminders were sent to participants who had not finished the survey, on either November 28th or 29th, and on December 6th 2024. No additional reminders were issued thereafter. The survey was officially closed to responses on December 31st, 2024.

Table S1.1. Item development and source information for QRP items, scientific norms and publication/funding pressure items.

	Survey Item Wording	Source of Item / Development Process	Source Item Wording
**General QRPs**			
QRP 1	Choosing references for citation in a way that primarily supports my own findings or beliefs.	Gopalakrishna et al. [14]	In the last three years, I selectively cited references to enhance my own findings or convictions.
QRP 2	Including individuals as authors on a paper who may not have contributed significantly to the research.	Schneider et al. [15]	Including authors on a publication who has not contributed sufficiently to the work to merit authorship.
QRP 3	Citing literature without having read it.	Schneider et al. [15]	Citing literature deemed relevant for your study without actually having read it
QRP 4	Making adjustments to the analysis, inferences or conclusions in a manuscript that you do not believe are valid in order to satisfy reviewers.	Original item developed in discussion with researchers and refined through piloting.	
QRP 5	Omitting citations of publications that present differing views or findings from one's own work.	Schneider et al. [15]	Deliberately disregard citing relevant publications that contradict own beliefs or research
QRP 6	Using content from papers under review for personal research or publication.	Original item developed in discussion with researchers and refined through piloting.	
QRP 7	Not disclosing relevant conflicts of interest.	Schneider et al. [15]	In a publication, failing to disclose relevant personal, financial, political or intellectual conflicts of interests.

QRP 8	Excluding junior researchers from the list of authors on a paper despite them having made significant contributions.	Original item developed in discussion with researchers and refined through piloting.	
**Quantitative QRPs**			
QRP 9	Neglecting to conduct a power analysis to determine the necessary sample size based on the intended significance level, statistical power, and anticipated effect size.	Original item developed in discussion with researchers and refined through piloting.	
QRP 10	Not fully disclosing and making accessible all underlying data, computer codes, or syntaxes used in research on open science platforms.	Adapated from Gopalakrishna et al. [14]	In the last three years, I contributed, where appropriate, to making my research data findable, accessible, interoperable and reusable in accordance with the FAIR principles.
QRP 11	Reporting a set of results as the complete set of analyses when other relevant analyses were also conducted.	John, Lowenstein & Prelec [1]	In a paper, failing to report all of a study's conditions
QRP 12	Presenting an unexpected finding or exploratory analysis in a research report as if it were an a priori hypothesis.	Schneider et al. [15]	Without disclosure, presenting an unexpected finding as having been predicted from the start.
QRP 13	Deciding not to report covariates that failed to reach statistical significance (e.g., $p < 0.05$) or some other desired statistical threshold.	John, Lowenstein & Prelec [1]	In a paper, selectively reporting studies that "worked"
QRP 14	Deciding to exclude data points after first checking the impact on statistical significance (e.g., $p < 0.05$) or some other desired statistical threshold.	John, Lowenstein & Prelec [1]	Deciding whether to exclude data after looking at the impact of doing so on the results
QRP 15	Rounding off p-values so they appear to be below or equal to the desired alpha (e.g., reporting $p = 0.054$ as $p = 0.05$).	John, Lowenstein & Prelec [1]	In a paper, "rounding off" a p value (e.g., reporting that a p value of .054 is less than .05)
QRP 16	Extending data collection or manipulating data selection during an analysis with the specific intent of obtaining statistically significant results where none were previously found.	Schneider et al. [15]	In significance testing studies, continue to collect more data in order to render non-significant results significant. For example: If initial results are not statistically significant, collect more data until you get the desired result.
**Qualitative QRPs**			
QRP 17	Not maintaining comprehensive and detailed documentation of all decisions, modifications, and steps undertaken throughout the research process.	Gopalakrishna et al. [14]	In the last three years, I kept a comprehensive record of my research decisions throughout my studies.
QRP 18	Not engaging in reflexivity, which involves failing to acknowledge or address my biases, perspectives, and potential influence on the research process.	Original item developed in discussion with qualitative researchers.	
QRP 19	Failing to sufficiently describe the methods of data collection and analysis, making it difficult for others to understand how conclusions were reached.	Original item developed in discussion with qualitative researchers.	
QRP 20	Phrasing questions in interviews or focus groups in a way that might influence participants towards certain responses.	Original item developed in discussion with qualitative researchers.	

QRP 21	Interpreting qualitative data in a way that confirms the analysts pre-existing ideas and beliefs, ignoring data that might suggest alternative interpretations.	Original item developed in discussion with qualitative researchers.	
QRP 22	Deliberately omitting or failing to discuss data that contradicts or does not align with the emerging themes or patterns identified in the research.	Original item developed in discussion with qualitative researchers.	
QRP 23	Presenting interview or focus group findings in a manner that may not fully reflect the original data.	Original item developed in discussion with qualitative researchers.	
QRP 24	Providing false or misleading information to participants about the purpose or nature of the study.	Original item developed in discussion with qualitative researchers.	
**Publication Pressure**			
	On a scale from 'very low pressure' to 'very high pressure', how much pressure do you feel there is to publish in your field?	Gopalakrishna et al. [14]	I feel pressure to publish.
	How would you rate your level of stress concerning your colleagues' evaluation of your publication output, on a scale from 'extremely stressful' to 'not stressful at all'?	Gopalakrishna et al. [14]	I experience stress at the thought of my colleagues' assessment of my publication output.
	To what extent do you agree that your colleagues judge you on the basis of your publications, on a scale from 'totally disagree' to 'totally agree'?	Gopalakrishna et al. [14]	I have the feeling that my colleagues judge me mainly on the basis of my publications.
	To what extent do you agree publication pressure harms your ability to do good research, on a scale from 'totally disagree' to 'totally agree'?	Gopalakrishna et al. [14]	Publication pressure harms my ability to do good research.
	To what extent do you agree that the current publication climate puts pressure on your relationships with fellow researchers, on a scale from 'totally disagree' to 'totally agree'?	Gopalakrishna et al. [14]	The current publication climate puts pressure on relationships with fellow-researchers.
	To what extent do you agree that publication pressure sometimes leads you to cut corners, on a scale from 'totally disagree' to 'totally agree'?	Gopalakrishna et al. [14]	Publication pressure sometimes leads me to cut corners.
	To what extent do you agree that your ability to obtain research funding depends on the number of publications you have, on a scale from 'totally disagree' to 'totally agree'?	Original item. The item was developed through discussions with researchers, a review of the literature, and pilot testing.	
**Funding Pressure**			
	To what extent do you agree that judgements of your academic performance are independent of your successful grant applications, on a scale from 'totally disagree' to 'totally agree'?	Gopalakrishna et al. [14]	Judgements of my academic performance are independent of my successful grant applications.
	To what extent do you agree your job security depends strongly on research grants you obtain, on a scale from 'totally disagree' to 'totally agree'?	Gopalakrishna et al. [14]	My job security depends strongly on research grants I receive.

	To what extent do you agree that your prospects for promotion depend on you obtaining funding, on a scale from 'totally disagree' to 'totally agree'?	Gopalakrishna et al. [14]	My prospects for promotion depend on me obtaining funding.
	To what extent do you agree that you would be able to do your research without obtaining your own funding, on a scale from 'totally disagree' to 'totally agree'?	Gopalakrishna et al. [14]	I would be able to do my research without obtaining my own funding.
	To what extent do you agree that obtaining your own research funding is crucial for your academic career, on a scale from 'totally disagree' to 'totally agree'?	Gopalakrishna et al. [14]	The continuation of my research depends on obtaining my own funding.
**Scientific Norms**			
Disinterestedness			
	The personal beliefs and values of researchers should play a role in the interpretation of data or outcomes in research.	Original item. The item was developed through discussions with researchers, a review of the literature, and pilot testing.	
	Researchers should consider modifying their areas of research interest to better align with available funding opportunities.	Macfarlane & Cheng [25]	I align my research interests with funding opportunities
	Researchers should manipulate research findings to benefit themselves, their research team, or their institution.	Original item. The item was developed through discussions with researchers, a review of the literature, and pilot testing.	
	Certain areas of research should be avoided because of their social and political implications.	Original item. The item was developed through discussions with researchers, a review of the literature, and pilot testing.	
	The novelty of research should be considered when deciding whether to pursue it.	Original item. The item was developed through discussions with researchers, a review of the literature, and pilot testing.	
Organised Scepticism			
	Researchers should aim to consider all new evidence, hypotheses, and theories, even those that challenge or contradict their own work.	Anderson, Martinson & De Vries [38]	Scientists consider all new evidence, hypotheses, theories, and innovations, even those that challenge or contradict their own work.
	Researchers should reject scientific findings solely because they contradict well-established knowledge.	Original item. The item was developed through discussions with researchers, a review of the literature, and pilot testing.	
	Researchers should disregard a scientific finding based on the author's affiliation with an institution that does not have widespread recognition	Original item. The item was developed through discussions with researchers, a review of	

		the literature, and pilot testing.	
	Researchers should periodically re-evaluate their own published findings, even in the absence of obvious errors, to ensure their conclusions remain valid and robust over time.	Original item. The item was developed through discussions with researchers, a review of the literature, and pilot testing.	
	Researchers should thoroughly scrutinise the methodology and inferences of a published study before making judgments about the validity and reliability of its findings.	Original item. The item was developed through discussions with researchers, a review of the literature, and pilot testing.	
Universalism			
	The personal background and characteristics of a researcher should be considered when assessing the validity of their scientific claims.	Original item. The item was developed through discussions with researchers, a review of the literature, and pilot testing.	
	The country where a researcher is based should influence a journal editor's decision to publish the researcher's work in their journal.	Original item. The item was developed through discussions with researchers, a review of the literature, and pilot testing.	
	Researchers should take into account the authors' ideological positions when evaluating the validity of the findings or conclusions presented in a scientific article.	Original item. The item was developed through discussions with researchers, a review of the literature, and pilot testing.	
	Researchers from any part of the world should have equal opportunities to collaborate and contribute to any scientific field.	Original item. The item was developed through discussions with researchers, a review of the literature, and pilot testing.	
	When assessing a job application, particular emphasis should be placed on a researcher's institutional affiliations.	Kim & Kim [26]	In order to be successful in the science community, it is important not only to be competent in research but to obtain a degree from a prestigious university
Communualism			
	It is fair for researchers to embargo data for a set period after data collection.	Bray D, von Storch H. [24]	Other scientists should have free access to my data after I have published the initial findings– absolutely not/under all circumstances
	Research findings should be a communal resource and not proprietary knowledge owned by individuals or institutions.	Bray D, von Storch H. [24]	Scientific results should be– private/public property
	Access to research findings should be managed through paywalls to ensure the sustainability and quality of scientific publishing.	Original item. The item was developed through discussions with researchers, a review of the literature, and pilot testing.	

	Researchers should withhold raw data and methodologies to prevent others from duplicating or challenging their work.	Original item. The item was developed through discussions with researchers, a review of the literature, and pilot testing.	
	Researchers should be obligated to upload data to public databases and repositories.	Original item. The item was developed through discussions with researchers, a review of the literature, and pilot testing.	

S1.2. Psychometrics

S1.2.1. *Publication and Funding Pressure*

Table S1.2. Confirmatory Factor Analysis full pattern matrix for publication pressure items

Item	Factor 1
To what extent do you agree that your ability to obtain research funding depends on the number of publications you have, on a scale from 'totally disagree' to 'totally agree'?	0.37
On a scale from 'very low pressure' to 'very high pressure', how much pressure do you feel there is to publish in your field?	0.47
How would you rate your level of stress concerning your colleagues' evaluation of your publication output, on a scale from 'extremely stressful' to 'not stressful at all'? (reverse coded)	0.37
To what extent do you agree that your colleagues judge you on the basis of your publications, on a scale from 'totally disagree' to 'totally agree'?	0.33
To what extent do you agree publication pressure harms your ability to do good research, on a scale from 'totally disagree' to 'totally agree'?	0.75
To what extent do you agree that the current publication climate puts pressure on your relationships with fellow researchers, on a scale from 'totally disagree' to 'totally agree'?	0.72
To what extent do you agree that publication pressure sometimes leads you to cut corners, on a scale from 'totally disagree' to 'totally agree'?	0.48
Note. χ^2(df) = 331.37 (14); CFI = 0.9; TLI = 0.86; RMSEA = 0.09	

Table S1.3. Confirmatory Factor Analysis full pattern matrix for funding pressure items

Item	Factor 1
To what extent do you agree that judgements of your academic performance are independent of your successful grant applications, on a scale from 'totally disagree' to 'totally agree'? (reverse coded)	0.24
To what extent do you agree your job security depends strongly on research grants you obtain, on a scale from 'totally disagree' to 'totally agree'?	0.65
To what extent do you agree that your prospects for promotion depend on you obtaining funding, on a scale from 'totally disagree' to 'totally agree'?	0.79
To what extent do you agree that you would be able to do your research without obtaining your own funding, on a scale from 'totally disagree' to 'totally agree'? (reverse coded)	0.43
To what extent do you agree that obtaining your own research funding is crucial for your academic career, on a scale from 'totally disagree' to 'totally agree'?	0.76
Note. χ^2(df) = 299.87 (5); CFI = 0.91 ; TLI = 0.83 ; RMSEA = 0.14	

Table S1.4. Mean scores and question wording for publication and funding pressure items, stratified by disciplinary field, career stage and sex.

Item Wording	Response Scale	Disciplinary Field				Career Stage				Sex		Full Sample
**Publication Pressure**		**Social Sciences**	**Natural Sciences**	**Medical Sciences**	**Arts and Humanities**	**Early-Career**	**Mid-Career**	**Late-career**	**Retired**	**Male**	**Female**	
On a scale from 'very low pressure' to 'very high pressure', how much pressure do you feel there is to publish in your field?	1 (Very Low Pressure) - 7 (Very High Pressure)	5.66	5.6	5.34	5.48	5.79	5.65	5.46	5.05	5.4	5.78	5.56
How would you rate your level of stress concerning your colleagues' evaluation of your publication output, on a scale from 'extremely stressful' to 'not stressful at all'? [Reverse Coded]	1 (Extremely Stressful) - 7 (Not Stressful At All)	3.85	3.75	3.68	3.95	4.22	3.94	3.52	3.26	3.64	4.03	3.8
To what extent do you agree that your colleagues judge you on the basis of your publications, on a scale from 'totally disagree' to 'totally agree'?	1 (Totally Disagree) - 7 (Totally Agree)	4.84	4.81	4.61	4.85	4.73	4.74	4.91	4.83	4.76	4.84	4.8
To what extent do you agree publication pressure harms your	1 (Totally Disagree) - 7 (Totally Agree)	4.11	4.13	3.77	4.09	4.64	4.25	3.75	3.11	3.92	4.25	4.06

ability to do good research, on a scale from 'totally disagree' to 'totally agree'?												
To what extent do you agree that the current publication climate puts pressure on your relationships with fellow researchers, on a scale from 'totally disagree' to 'totally agree'?	1 (Totally Disagree) - 7 (Totally Agree)	4.01	4.06	3.89	3.99	4.34	4.17	3.71	3.56	3.88	4.18	4.01
To what extent do you agree that publication pressure sometimes leads you to cut corners, on a scale from 'totally disagree' to 'totally agree'?	1 (Totally Disagree) - 7 (Totally Agree)	2.64	2.62	2.42	2.54	3.06	2.73	2.3	1.86	2.6	2.57	2.58
To what extent do you agree that your ability to obtain research funding depends on the number of publications you have, on a scale from 'totally disagree' to 'totally agree'?	1 (Totally Disagree) - 7 (Totally Agree)	5.45	5.65	5.47	5.35	5.8	5.54	5.44	5.03	5.44	5.59	5.51
**Funding Pressure**												

To what extent do you agree that judgements of your academic performance are independent of your successful grant applications, on a scale from 'totally disagree' to 'totally agree'? [Reverse Coded]	1 (Totally Disagree) - 7 (Totally Agree)	4.55	4.77	4.59	4.37	4.61	4.72	4.56	4.24	4.56	4.66	4.6
To what extent do you agree your job security depends strongly on research grants you obtain, on a scale from 'totally disagree' to 'totally agree'?	1 (Totally Disagree) - 7 (Totally Agree)	3.57	4.02	3.79	3.55	4.88	3.85	3.06	3.25	3.57	4.01	3.76
To what extent do you agree that your prospects for promotion depend on you obtaining funding, on a scale from 'totally disagree' to 'totally agree'?	1 (Totally Disagree) - 7 (Totally Agree)	4.69	5.19	4.69	4.69	5.37	5.14	4.42	4.18	4.76	5.03	4.88
To what extent do you agree that you would be able to do your research without	1 (Totally Disagree) - 7 (Totally Agree)	3.89	4.67	4.56	3.63	4.58	4.3	4.16	3.48	4.1	4.4	4.23

obtaining your own funding, on a scale from 'totally disagree' to 'totally agree'? [Reverse Coded]												
To what extent do you agree that obtaining your own research funding is crucial for your academic career, on a scale from 'totally disagree' to 'totally agree'?	1 (Totally Disagree) - 7 (Totally Agree)	5.01	5.58	5.23	4.85	5.8	5.36	4.97	4.32	5.07	5.43	5.23

S1.2.2. ***Scientific Norms***

The initial scientific norms item pool consisted of 20 items, with five items corresponding to each Mertonian norm. I first examined the factorability of the full 20 item pool. Following iterative exploratory factor analyses, items with weak, cross-loading, or theoretically incoherent loadings were removed. The final retained solution comprised 10 items loading onto three interpretable factors: universalism, communalism, and organised scepticism. The pattern matrix for the final retained 10 item solution is shown in Table S1.5. Items were removed if they failed to load ≥ .30 on any factor, showed substantial cross-loadings, or did not align theoretically with the emerging factor. The final retained solution prioritised interpretability, simple structure, and alignment with Mertonian norms. For the initial 20 item pool, the Kaiser-Meyer-Olkin (KMO) measure of sampling adequacy was 0.76, indicating strong factorability. Bartlett's test of sphericity was significant ($X^2 = 6075.19(190)$, $p < 0.05$), confirming sufficient correlations among items for factor analysis. Maximum likelihood factor extraction with oblimin rotation was used.

The internal consistency for each factor was lower than conventional thresholds and the Cronbach's alpha threshold of 0.7 included in our pre-registration ($\alpha = 0.48 – 0.59$; see Table 5), but the factors were retained based on theoretical importance and the distinct clustering present in the factor structure (that is, items clearly load onto distinct factors). The lack of internal consistency likely suggests that adherence to these norms is not uniform and unidimensional, potentially reflecting shifts in contemporary research practices rather than measurement error. Moreover, the small Cronbach's alpha may result from the small number of items, as Cronbach's alpha is sensitive to scale length.

Table S1.5. Exploratory Factor Analysis full pattern matrix for scientific norm items

Item	Factor 1 Universalism	Factor 2 Communalism	Factor 3 Organised Scepticism	Communalities
The personal background and characteristics of a researcher should be considered when assessing the validity of their scientific claims. (Reversed)	**0.669**	0.009	0.001	0.449
The country where a researcher is based should influence a journal editor's decision to publish the researcher's work in their journal. (Reversed)	**0.447**	-0.003	0.109	0.231
Researchers should take into account the authors' ideological positions when evaluating the validity of the findings or conclusions presented in a scientific article. (Reversed)	**0.609**	-0.017	-0.079	0.359
The personal beliefs and values of researchers should play a role in the interpretation of data or outcomes in research. (Reversed)	**0.364**	0.034	0.122	0.171
Researchers should aim to consider all new evidence, hypotheses, and theories, even those that challenge or contradict their own work.	-0.004	-0.011	**0.667**	0.441
Researchers should reject scientific findings solely because they contradict well-established knowledge. (Reversed)	0.090	-0.042	**0.372**	0.152
Researchers should thoroughly scrutinise the methodology and inferences of a published study before making judgments about the validity and reliability of its findings.	-0.041	0.037	**0.492**	0.246
Research findings should be a communal resource and not proprietary knowledge owned by individuals or institutions.	-0.005	**0.411**	0.103	0.197
Researchers should be obligated to upload data to public databases and repositories.	-0.013	**0.708**	-0.043	0.494
Researchers should withhold raw data and methodologies to prevent others from duplicating or challenging their work. (Reversed)	0.111	**0.370**	0.192	0.239
**SS Loading**	1.176	0.908	0.815	
**% Variance**	0.118	0.091	0.082	

Table reports the final 10 item, three-factor solution after item reduction. Loadings ≥ .30 are bolded. Extraction used maximum likelihood with oblimin rotation. Communalities and sums of squared loadings are reported.

S1.2.3. ***Personal and Proximal Scientific Motivations and Scientific Approach***

Eighteen items were used to measure motivations and epistemic orientation. An exploratory factor analysis identified six factors: two capturing personal motivations, two capturing science-specific motivations, and two capturing epistemic orientation. The Kaiser-Meyer-Olkin measure of sampling adequacy was 0.80, suggesting strong factorability. Bartlett’s test of sphericity was significant ($X^2 = 21032.87(153)$, $p < 0.05$) indicating that items are sufficiently correlated to justify factor analysis. A six-factor solution was extracted based on eigenvalues, factor loadings and theoretical justification. Because an oblique rotation was used, pattern coefficients may exceed 1.0.The full pattern matrix is shown in Table S1.7.

The two personal motivation factors comprised a ‘career growth’ motivation, reflecting a drive for career growth and security, and an ‘ego and esteem’ motivation, reflecting a desire to obtain fame, avoid external perceptions of failure and achieve political or social goals. The two proximal science-specific motivations comprised a motivation for ‘impact

and quality', reflecting a drive for scientific and societal impact, and a drive for 'academic/scientific achievement', reflecting a motivation to improve one's scientific success (e.g., number of publications, citation index).

Impact and quality motivation and ego and esteem motivation exhibited lower reliability, with a Cronbach's α of 0.52 and 0.58 respectively. This likely indicates some heterogeneity in these motivations or overlapping dimensions not fully captured by the factor structure. Career growth motivation and achievement motivation demonstrated good internal consistency (α = 0.74 and 0.83, respectively), supporting their validity as distinct and reliable constructs. A clear 'falsification' vs. 'confirmation' distinction emerged, with both orientations showing strong internal consistency (α = 0.83 and 0.81, respectively). Falsification orientation reflects an emphasis on critically testing and potentially refuting theories and findings, including one's own. Confirmation orientation reflects a preference for reinforcing one's previous research and theories.

The retained factors accounted for approximately 51% of total variance, with individual factor contributions ranging from 5% to 11.6%. Items loading onto each factor were averaged to create composite scores, computing a mean score for each respondent for each of the motivations and orientations. Higher scores indicated greater motivation or stronger orientation. These scales were standardised to z-scores, ensuring each score is mean-centered and scaled by the standard deviation.

Table S1.6. Mean scores and question wording for personal and proximal-science specific items, stratified by disciplinary field, career stage and sex.

**Item Wording**	**Disciplinary Field**				**Career Stage**				**Sex**		**Full Sample**
**Personal Motivations**	**Social Sciences**	**Natural Sciences**	**Medical Sciences**	**Arts and Humanities**	**Early-Career**	**Mid-Career**	**Late-career**	**Retired**	**Male**	**Female**	
Advancing my career or achieving promotion, compared to other personal and professional motivations	3.5	3.43	3.59	3.54	3.77	3.55	3.48	2.81	3.41	3.6	3.49
Securing financial benefits (e.g., an increase in pay) or rewards for myself, compared to other personal and professional motivations	3.3	3.14	3.14	3.28	3.63	3.31	3.11	2.32	3.1	3.37	3.21
Ensuring my job security or reducing the risk of job loss, compared to other personal and professional motivations	3.73	3.66	3.44	3.83	4.4	3.92	3.31	2.4	3.49	3.94	3.68

Obtaining respect or fame, compared to other personal and professional motivations	2.91	2.81	2.95	3.03	2.62	2.85	3.06	3.17	2.91	2.89	2.9
Avoiding being perceived as a failure or unsuccessful in my field, compared to other personal and professional motivations	3.4	3.42	3.23	3.5	3.52	3.4	3.41	3.12	3.28	3.57	3.4
Achieving my social or political goals, compared to other personal and professional motivations	2.83	2.16	2.58	2.74	2.71	2.57	2.38		2.36	2.75	2.53
**Proximal Science-Specific Motivations**											
Obtaining a higher citation index	3.03	3.15	3.34	2.47	3.2	3.11	2.9	2.67	3	3.05	3.02
Increasing my number of publications	4.06	3.9	4	3.81	4.15	4	3.93	3.41	3.9	4.02	3.95
Increasing my opportunities for research funding	3.78	4.12	4	3.7	4.31	4.07	3.86	2.76	3.75	4.15	3.92
Producing new and exciting research findings	5.15	5.35	5.15	5.28	5.18	5.25	5.33	5.13	5.22	5.28	5.25
Maximising the impact of my research outside of academia<br><br>Clarification: by 'impact outside of academia' we mean effecting change or benefit to society, culture, the economy, public policy or services, health, the environment or quality of life.	4.59	4.22	4.67	4.22	4.45	4.53	4.3	4.05	4.18	4.69	4.4

Ensuring the reproducibility and accuracy of my research findings	4.25	4.71	4.75	4.25	4.36	4.52	4.53	4.54	4.44	4.57	4.49
Increasing my likelihood of publication in a prestigious outlet	4.07	3.90	4.00	3.85	4.16	4.00	3.93	3.46	3.90	4.03	3.96
**Scientific Approach**											
Confirming my past findings	2.91	3.45	3.54	3.2	3.26	3.15	3.29	3.52	3.19	3.33	3.25
Disconfirming my past findings	2.33	2.58	2.76	2.43	2.48	2.47	2.51	2.64	2.58	2.39	2.5
Disconfirming my own theories	2.47	2.71	2.98	2.51	2.59	2.59	2.68	2.8	2.72	2.52	2.64
Disconfirming competing theories or findings	3.16	3.31	3.41	3.47	3.31	3.21	3.37	3.52	3.41	3.17	3.31
Confirming my own theories	3.28	3.80	3.85	3.66	3.76	3.52	3.61	3.81	3.62	3.63	3.62

Table S1.7. Exploratory factor analysis full pattern matrix for personal, proximal-scientific and scientific motivation items

	**Factor 1 Achievement Motivation (Proximal Science-Specific)**	**Factor 2 Falsification Orientation (Approach)**	**Factor 3 Career Growth Motivation (Personal)**	**Factor 4 Confirmation Orientation (Approach)**	**Factor 5 Ego & Esteem Motivation (Personal)**	**Factor 6 Impact & Quality Motivation (Proximal Science-Specific)**	**Communalities**
Obtaining a higher citation index	**0.681**	0.002	0.023	0.096	0.074	-0.061	**0.546**
Increasing my number of publications	**0.855**	-0.008	0.000	-0.001	-0.029	-0.065	**0.687**
Increasing my likelihood of publication in a prestigious outlet	**0.766**	-0.017	0.018	-0.003	0.027	0.103	**0.673**
Increasing my opportunities for research funding	**0.437**	0.031	0.275	-0.041	-0.061	0.226	**0.473**
Disconfirming my past findings	-0.050	**0.778**	0.036	0.148	-0.014	-0.012	**0.761**
Disconfirming my own theories	0.008	**1.029**	-0.007	-0.069	-0.010	0.003	**0.973**
Disconfirming competing theories or findings	0.081	**0.446**	-0.048	0.136	0.121	0.059	**0.341**
Advancing my career or achieving promotion, compared to other personal and professional motivations	0.126	-0.003	**0.634**	-0.009	0.101	0.039	**0.558**
Securing financial benefits (e.g., an	-0.023	0.014	**0.837**	0.022	-0.007	-0.066	**0.659**

increase in pay) or rewards for myself, compared to other personal and professional motivations							
Ensuring my job security or reducing the risk of job loss, compared to other personal and professional motivations	0.016	-0.029	**0.567**	0.005	-0.071	0.043	**0.347**
Confirming my past findings	-0.005	-0.010	0.029	**0.999**	-0.005	-0.004	**0.995**
Confirming my own theories	0.121	0.199	-0.057	**0.539**	0.063	0.059	**0.521**
Achieving my social or political goals, compared to other personal and professional motivations	-0.119	-0.016	0.217	-0.023	**0.345**	0.112	**0.182**
Obtaining respect or fame, compared to other personal and professional motivations	0.006	-0.003	-0.003	0.001	**0.998**	-0.001	**0.995**
Avoiding being perceived as a failure or unsuccessful in my field, compared to other personal and professional motivations	0.209	0.017	0.099	-0.023	**0.311**	0.033	**0.243**
Producing new and exciting research findings	0.047	-0.014	-0.060	-0.011	0.018	**0.622**	**0.413**
Maximising the impact of my research outside of academia	-0.098	0.025	0.158	-0.002	0.051	**0.422**	**0.206**
Ensuring the reproducibility and accuracy of my research findings	-0.034	0.074	-0.048	0.243	-0.038	**0.477**	**0.358**
SS Loading	**2.085**	**1.912**	**1.6597**	**1.406**	**1.262**	**0.893**	
% Variance	**0.116**	**0.106**	**0.089**	**0.078**	**0.070**	**0.050**	

Factor loadings above 0.30 are emboldened for clarity.
EFA was conducted using maximum likelihood extraction with oblimin rotation.
Retained items for each factor emboldened.
Communalities (h^2) and SS loadings for each factor are provided to indicate the proportion of variance explained.

Table S1.8. Mapping pre-registered hypothesis and analysis to the final reported models

Pre-registered Hypothesis / Analysis	Pre-registered Status	Corresponding Manuscript Hypothesis	How handled in final manuscript	Reason for change/note
**H1:** Researchers who are more agreeable and conscientious engage in fewer QRPs on average.	Confirmatory	H19a and H19b	Retained	Renumbered
**H2:** The greater the perceived pressure to publish, the greater the engagement in QRPs.	Confirmatory	H9	Retained	Renumbered
**H3:** The greater the perceived pressure to obtain funding, the greater the engagement in QRPs.	Confirmatory	H10	Retained	Renumbered
**H4-H7:** The more a researcher is motivated by a) maximising career rewards, b) ensuring job security, c) achieving social or political goals, or d) obtaining fame or social approbation, the greater their engagement in QRPs.	Confirmatory	H1 & H2	Modified / consolidated	Original personal-motivation categories were empirically consolidated through factor analysis into broader factors, principally **career growth** and **ego and esteem**
**H8-H10:** The more a researcher is motivated by a) increasing research output (e.g., increase publication counts, citation metrics, obtaining funding etc.), b) maximising novelty of research and c) maximising research impact, the greater their engagement in QRPs.	Confirmatory	H3 & H4	Modified / partially retained	Final proximal motivation factors were consolidated into **achievement motivation** and **impact and quality motivation**; **novelty** was not retained as a distinct reported factor
**H11-H28:** The relationship between the personal motivations of a) maximising career rewards, b) ensuring job security, and c) obtaining fame or social approbation, and the proximal science-specific motivations of a) increasing research output, b) achieving novelty and c) maximising research impact, are stronger when there is greater perceived pressure to a) publish and b) increase funding.	Confirmatory	H11-H18	Retained in simplified form	Original preregistration specified a larger grid of personal × proximal pathways; final manuscript reports a condensed factor-based version of these moderation tests
**H29-H37:** The relationship between the personal motivations of a) maximising career rewards, b) ensuring job security, c) achieving social approbation or fame, and increased QRP engagement are mediated by a) increasing research output, b) achieving novelty and c) maximising research impact.	Confirmatory	H5 – H8	Retained in simplified form	Original mediation hypotheses were condensed after factor development and estimated using a narrower SEM with empirically derived factors
**H38:** The relationship between the personal motivations of achieving social and political goals, and QRPs, is mediated by the proximal science-specific motivation increasing research output.	Confirmatory	Included indirectly within SEM involving ego/esteem and achievement	Modified / absorbed	Social/political goals were not retained as a standalone predictor; they were folded into the broader **ego and esteem** factor

Use two dependent variables: overall mean QRP and any frequent QRP.	Confirmatory	Mean QRP & any frequent QRP	Retained	Same two outcomes used in manuscript
Conduct EFA on personal motivations, proximal motivations, and scientific norms; create composite scores based on identified factors.	Confirmatory / analytic plan	Factor development described in Methods	Retained, but resulting factors differed from anticipated labels	Final factors reflected empirical structure rather than all preregistered labels; this affected one-to-one hypothesis mapping
Scientific norms included as exploratory predictors.	Exploratory	Universalism, Communalism, Organised scepticism analyses	Retained / extended	Final manuscript reports disaggregated norm factors; **disinterestedness** did not emerge as a distinct retained factor
Explore falsification, confirmation, and reproducibility-related orientation.	Exploratory	Falsification and confirmation analyses	Partially retained / modified	Falsification and confirmation were retained; reproducibility was not reported as a distinct final factor/scale in the current manuscript framing
Linear and logistic regressions for Average QRP and Any Frequent QRP with demographic controls.	Confirmatory / analytic plan	Models 1A–2B	Retained	Same broad strategy used
Moderation analyses via hierarchical regression with interaction terms.	Confirmatory / analytic plan	Models 3A–3B and additional exploratory interactions	Retained, with extensions	Core pressure * motivation moderation tests retained; added exploratory pressure * norms and pressure * personality interactions beyond core preregistration
Mediation analysis using SEM; direct, indirect, total effects to be reported.	Confirmatory / analytic plan	Models 4A–4C / final SEM	Retained, but simplified	Final SEM treated variables as manifest and used factor-derived constructs; manuscript now presents final model in main text and earlier specifications in Tables S2.7 to S2.9
Further analyses not identified above are exploratory in nature.	Exploratory	Political orientation, additional interaction terms, some norm-specific analyses	Retained as exploratory	Explicitly labelled as exploratory in manuscript

S2. Supplementary Results

S2.1. Descriptive Statistics

Table S2.1. Proportion of respondents selecting each answer category for each QRP item (*N* = 3050)

	Item Wording	Methodological Group	Never (%, 95% CI)	Rarely (%, 95% CI)	Sometimes (%, 95% CI)	Often (%, 95% CI)	Always (%, 95% CI)
**QRP 1**	Choosing references for citation in a way that primarily supports	Non-empirical	43.4 (41.4, 45.4)	27.3 (25.5, 29.1)	21.5 (19.8, 23.2)	6.6 (5.6, 7.6)	1.2 (0.7, 1.6)

	my own findings or beliefs.						
**QRP 2**	Including individuals as authors on a paper who may not have contributed significantly to the research.	Non-empirical	43.8 (41.8, 45.9)	27.3 (25.5, 29.1)	20.6 (18.9, 22.2)	6.8 (5.8, 7.9)	1.5 (0.9, 2)
**QRP 3**	Citing literature without having read it.	Non-empirical	45.3 (43.3, 47.3)	35.3 (33.4, 37.2)	15.9 (14.4, 17.3)	3.3 (2.6, 4)	0.3 (0.1, 0.5)
**QRP 4**	Making adjustments to the analysis, inferences or conclusions in a manuscript that you do not believe are valid in order to satisfy reviewers.	Non-empirical	68.6 (66.7, 70.5)	21.4 (19.7, 23.1)	7.9 (6.9, 9)	1.7 (1.2, 2.3)	0.4 (0.1, 0.6)
**QRP 5**	Omitting citations of publications that present differing views or findings from one's own work.	Non-empirical	78.3 (76.6, 79.9)	18 (16.4, 19.5)	3.2 (2.5, 4)	0.4 (0.1, 0.6)	0.2 (0, 0.3)
**QRP 6**	Using content from papers under review for personal research or publication.	Non-empirical	88.2 (86.8, 89.5)	7.5 (6.4, 8.6)	3.3 (2.5, 4)	0.8 (0.5, 1.2)	0.2 (0, 0.4)
**QRP 7**	Not disclosing relevant conflicts of interest.	Non-empirical	96 (95.1, 96.8)	2.6 (2, 3.3)	0.8 (0.4, 1.2)	0.3 (0.1, 0.6)	0.2 (0, 0.5)
**QRP 8**	Excluding junior researchers from the list of authors on a paper despite them having made significant contributions.	Non-empirical	98.5 (98, 99)	1 (0.6, 1.4)	0.4 (0.1, 0.6)	0.2 (0, 0.4)	0 (0, 0)
**QRP 9**	Neglecting to conduct a power analysis to determine the necessary sample size based on the intended significance level, statistical power, and anticipated effect size.	Quantitative	40 (37.1, 42.9)	26.1 (23.5, 28.7)	19.1 (16.8, 21.4)	10.7 (8.9, 12.6)	4.1 (2.9, 5.2)
**QRP 10**	Not fully disclosing and making accessible all underlying data, computer codes, or syntaxes used in research on open science platforms.	Quantitative	44.4 (41.7, 47.1)	20.2 (18, 22.4)	19.7 (17.5, 21.9)	11.8 (10, 13.5)	3.9 (2.9, 5)
**QRP 11**	Reporting a set of results as the complete set of analyses when other relevant analyses were also conducted.	Quantitative	59.7 (57.2, 62.3)	22.4 (20.3, 24.6)	12 (10.3, 13.7)	4 (3, 5)	1.9 (1.2, 2.6)
**QRP 12**	Presenting an unexpected finding or exploratory analysis in	Quantitative	66.2 (63.7, 68.8)	18.5 (16.4, 20.5)	10.9 (9.3, 12.6)	2.8 (1.9, 3.7)	1.5 (0.9, 2.2)

	a research report as if it were an a priori hypothesis.						
**QRP 13**	Deciding not to report covariates that failed to reach statistical significance (e.g., $p < 0.05$) or some other desired statistical threshold.	Quantitative	66.5 (63.9, 69.1)	19.4 (17.2, 21.6)	10 (8.3, 11.7)	2.5 (1.7, 3.4)	1.6 (0.9, 2.3)
**QRP 14**	Deciding to exclude data points after first checking the impact on statistical significance (e.g., $p < 0.05$) or some other desired statistical threshold.	Quantitative	78.4 (76.3, 80.6)	13.4 (11.6, 15.2)	6.3 (5, 7.6)	1.2 (0.6, 1.7)	0.7 (0.3, 1.2)
**QRP 15**	Rounding off p-values so they appear to be below or equal to the desired alpha (e.g., reporting $p = 0.054$ as $p = 0.05$).	Quantitative	87.1 (85.3, 88.9)	6.8 (5.5, 8.2)	4.2 (3.1, 5.2)	1.6 (0.9, 2.3)	0.3 (0, 0.6)
**QRP 16**	Extending data collection or manipulating data selection during an analysis with the specific intent of obtaining statistically significant results where none were previously found.	Quantitative	87.6 (85.9, 89.3)	7.7 (6.3, 9.1)	3.9 (2.9, 4.9)	0.6 (0.2, 0.9)	0.2 (0, 0.5)
**QRP 17**	Not maintaining comprehensive and detailed documentation of all decisions, modifications, and steps undertaken throughout the research process.	Qualitative	43.8 (40.8, 46.9)	31.6 (28.8, 34.5)	18 (15.6, 20.3)	5.8 (4.4, 7.3)	0.8 (0.2, 1.3)
**QRP 18**	Not engaging in reflexivity, which involves failing to acknowledge or address my biases, perspectives, and potential influence on the research process.	Qualitative	52.5 (49.5, 55.6)	35 (32.1, 37.9)	10.1 (8.3, 12)	1.8 (1, 2.6)	0.6 (0.1, 1.1)
**QRP 19**	Failing to sufficiently describe the methods of data collection and analysis, making it difficult for others to understand how conclusions were reached.	Qualitative	66.4 (63.6, 69.2)	24 (21.5, 26.5)	8.2 (6.5, 9.8)	1.2 (0.6, 1.9)	0.2 (0, 0.5)
**QRP 20**	Phrasing questions in interviews or focus groups in a way that	Qualitative	66.7 (63.4, 69.9)	25.6 (22.6, 28.6)	6.6 (4.9, 8.3)	0.9 (0.2, 1.5)	0.3 (0, 0.6)

	might influence participants towards certain responses.					
**QRP 21**	Interpreting qualitative data in a way that confirms the analysts pre-existing ideas and beliefs, ignoring data that might suggest alternative interpretations.	Qualitative	75.4 (72.7, 78.1)	20.4 (17.9, 22.9)	3.7 (2.5, 4.9)	0.2 (0, 0.5)	0.3 (0, 0.6)
**QRP 22**	Deliberately omitting or failing to discuss data that contradicts or does not align with the emerging themes or patterns identified in the research.	Qualitative	80.2 (77.8, 82.7)	16 (13.8, 18.3)	3.1 (2, 4.1)	0.6 (0.1, 1.1)	0.1 (0, 0.3)
**QRP 23**	Presenting interview or focus group findings in a manner that may not fully reflect the original data.	Qualitative	81.3 (78.7, 84)	14.4 (12, 16.8)	2.8 (1.7, 4)	1 (0.3, 1.7)	0.4 (0, 0.9)
**QRP 24**	Providing false or misleading information to participants about the purpose or nature of the study.	Qualitative	95.3 (93.9, 96.6)	2.8 (1.8, 3.9)	1.3 (0.6, 2.1)	0.3 (0, 0.7)	0.2 (0, 0.5)

Notes. Percentages are weighted; 95% Wald confidence intervals in brackets, computed with a simple weighted design.

S2.2. Bivariate Correlations

Table S2.2. Bivariate correlations between all variables

Variable	Mean QRP	Any frequent QRP	Universalism	Organised scepticism	Communalism	Achievement motivation	Falsification orientation	Career growth motivation	Confirmation orientation	Ego and esteem motivation	Impact and quality motivation	Funding pressure	Publication pressure	Extraversion	Agreeableness	Conscientiousness	Emotional stability	Openness	Female	Medical sciences	Social sciences	Humanities	Early career	Late career	Retired	Qualitative	Mixed methods	USA	Other country	Political orientation
**Mean QRP**	1.00																													
**Any frequent QRP**	0.57	1.00																												
**Universalism**	-0.17	-0.03	1.00																											
**Organised scepticism**	-0.24	-0.08	0.16	1.00																										
**Communalism**	-0.10	-0.04	0.14	0.20	1.00																									
**Achievement motivation**	0.13	0.08	0.04	-0.04	-0.10	1.00																								
**Falsification orientation**	-0.07	-0.07	0.02	0.07	0.12	0.07	1.00																							
**Career growth motivation**	0.09	0.04	0.03	-0.04	-0.10	0.50	0.05	1.00																						

**Confirmation orientation**	-0.01	-0.02	0.04	-0.01	-0.03	0.29	0.58	0.16	1.00																					
**Ego and esteem motivation**	0.14	0.04	-0.10	-0.03	-0.09	0.38	0.08	0.33	0.18	1.00																				
**Impact and quality motivation**	-0.14	-0.05	0.06	0.12	0.06	0.23	0.27	0.12	0.34	0.18	1.00																			
**Funding pressure**	0.08	0.09	0.08	0.02	0.09	0.30	0.04	0.29	0.07	0.06	0.11	1.00																		
**Publication pressure**	0.18	0.13	0.00	-0.02	0.03	0.29	0.04	0.29	0.09	0.15	0.08	0.42	1.00																	
**Extraversion**	-0.02	-0.03	-0.01	0.01	-0.00	0.07	0.05	0.02	0.03	0.09	0.12	-0.02	-0.05	1.00																
**Agreeableness**	-0.11	-0.07	0.05	0.07	0.01	-0.03	-0.06	-0.00	-0.01	-0.02	0.07	-0.03	-0.05	0.05	1.00															
**Conscientiousness**	-0.18	-0.04	0.06	0.16	-0.01	0.00	-0.01	0.03	0.04	0.00	0.14	-0.04	-0.11	0.00	0.14	1.00														
**Emotional stability**	-0.19	-0.10	0.04	0.06	-0.00	-0.06	0.03	-0.09	0.04	-0.09	0.07	-0.11	-0.24	0.06	0.22	0.26	1.00													
**Openness**	-0.17	-0.08	0.04	0.12	0.06	0.02	0.11	-0.01	0.10	0.07	0.27	0.00	-0.06	0.27	0.05	0.15	0.15	1.00												
**Female**	-0.02	-0.00	0.01	0.08	-0.10	0.08	-0.08	0.11	0.03	0.08	0.13	0.11	0.11	0.11	0.14	0.11	-0.08	0.02	1.00											
**Medical sciences**	0.01	0.02	-0.04	0.06	-0.02	0.04	0.06	-0.03	0.07	-0.01	0.06	0.01	-0.07	0.02	0.03	0.06	0.10	-0.00	0.03	1.00										
**Social sciences**	0.04	0.04	-0.10	-0.01	-0.06	0.01	-0.08	0.02	-0.15	0.05	-0.04	-0.10	0.02	0.05	0.01	0.00	-0.03	-0.05	0.09	-0.27	1.00									
**Humanities**	-0.09	-0.11	-0.11	0.05	-0.02	-0.08	0.01	0.04	0.00	0.05	-0.06	-0.11	-0.01	0.03	0.08	0.06	-0.01	0.09	0.06	-0.19	-0.31	1.00								
**Early-career**	0.13	0.09	0.04	-0.05	0.01	0.10	-0.01	0.18	0.02	0.01	-0.03	0.19	0.16	-0.05	-0.03	-0.07	-0.10	-0.09	0.06	0.02	-0.01	-0.04	1.00							
**Late-career**	-0.11	-0.09	-0.02	0.04	0.00	-0.04	0.02	-0.08	0.01	0.01	0.01	-0.16	-0.12	0.06	0.03	0.06	0.07	0.08	-0.04	-0.01	0.01	0.02	-0.32	1.00						
**Retired**	-0.05	-0.05	-0.09	0.01	0.02	-0.16	0.05	-0.22	0.06	-0.01	-0.05	-0.16	-0.17	0.02	0.02	0.04	0.07	0.07	-0.11	-0.01	-0.01	0.02	-0.16	-0.21	1.00					
**Qualitative**	-0.03	-0.09	-0.15	0.00	-0.11	-0.07	-0.06	-0.02	-0.13	0.08	-0.09	-0.13	-0.00	0.05	0.06	0.02	-0.02	0.03	0.10	-0.11	0.15	0.25	-0.02	0.03	0.02	1.00				
**Mixed methods**	-0.04	-0.03	-0.02	-0.01	-0.04	0.03	0.04	0.04	0.13	0.02	0.10	0.02	0.02	0.07	-0.01	0.01	0.03	0.08	0.02	-0.02	-0.15	0.06	-0.01	0.00	0.02	-0.45	1.00			
**USA**	-0.02	0.00	-0.04	0.01	-0.01	-0.08	-0.04	0.01	-0.04	-0.01	-0.05	-0.10	-0.08	-0.02	0.06	0.03	0.01	-0.05	-0.01	-0.04	0.04	0.06	-0.04	0.08	0.06	0.04	-0.06	1.00		
**Other country**	-0.03	-0.02	-0.11	-0.00	-0.01	-0.07	-0.02	-0.04	-0.05	0.03	0.04	-0.01	-0.03	-0.00	0.07	0.06	0.04	0.03	0.01	0.06	0.04	-0.03	-0.05	0.04	0.07	0.08	-0.02	-0.08	1.00	
**Political orientation**	0.01	0.02	0.03	-0.11	-0.20	0.14	0.02	0.09	0.12	-0.02	-0.00	-0.03	-0.01	-0.02	-0.05	0.02	0.06	-0.10	-0.06	0.05	-0.03	-0.11	-0.05	0.05	-0.01	-0.09	0.05	-0.05	-0.04	1.00

S2.3. Analysis Results under MNAR Sensitivity Analysis

Table S2.3. Regression Coefficients (95% CI) of mean QRP and frequent QRP by explanatory variables under MNAR-moderate assumption

	**Overall Mean QRP**		**Any Frequent QRP**	
	**Linear Regression Model 1A Coefficient (95% CI)**	**Linear Regression Model 1B Coefficient (95% CI)**	**Logistic Regression Model 2A OR (95% CI)**	**Logistic Regression Model 2B OR (95% CI)**
**Publication Pressure**	0.12 (0.08, 0.16)***	0.12 (0.08, 0.16)***	1.24 (1.12, 1.37)***	1.24 (1.12, 1.38)***
**Funding Pressure**	-0.01 (-0.05, 0.03)	-0.01 (-0.05, 0.03)	1.05 (0.94, 1.16)	1.04 (0.94, 1.16)
**Scientific Norms**				
Universalism	-0.14 (-0.18, -0.10)***	-0.14 (-0.18, -0.1)***	0.9 (0.82, 0.99)*	0.92 (0.83, 1.01)
Communalism	-0.04 (-0.07, 0.00)	-0.04 (-0.08, 0)	0.93 (0.85, 1.02)	0.93 (0.84, 1.02)
Organised Scepticism	-0.16 (-0.21, -0.11)***	-0.16 (-0.21, -0.11)***	0.91 (0.83, 0.99)*	0.90 (0.82, 0.98)*
**Personal Motivations**				
Career Growth	-0.03 (-0.07, 0.01)	-0.03 (-0.07, 0.01)	0.9 (0.81, 1.01)	0.9 (0.8, 1.00)
Ego and Esteem	0.11 (0.07, 0.15)***	0.11 (0.07, 0.15)***	1.08 (0.97, 1.19)	1.07 (0.97, 1.19)
**Proximal Science-Specific Motivations**				
Impact and Quality	-0.11 (-0.15, -0.07)***	-0.11 (-0.15, -0.07)***	0.91 (0.82, 1.01)	0.91 (0.82, 1.01)
Achievement	0.07 (0.03, 0.11)***	0.07 (0.03, 0.11)***	1.12 (1.00, 1.26)	1.13 (1.00, 1.27)*
**Research Approach**				
Falsification	-0.06 (-0.11, -0.01)*	-0.06 (-0.11, -0.01)*	0.86 (0.76, 0.97)**	0.86 (0.76, 0.97)**
Confirmation	0.03 (-0.02, 0.08)	0.03 (-0.02, 0.08)	1.02 (0.90, 1.16)	1.03 (0.91, 1.16)
**Personality Traits**				
Extraversion	0.03 (-0.01, 0.06)	0.03 (-0.01, 0.06)	1.02 (0.93, 1.12)	1.02 (0.93, 1.12)
Agreeableness	-0.03 (-0.07, 0.00)*	-0.03 (-0.07, 0.00)*	0.9 (0.82, 0.99)*	0.90 (0.82, 0.99)*
Conscientiousness	-0.07 (-0.10, -0.03)***	-0.07 (-0.11, -0.03)***	1.05 (0.96, 1.15)	1.05 (0.95, 1.16)
Emotional Stability (Neuroticism)	-0.08 (-0.12, -0.04)***	-0.08 (-0.11, -0.04)***	0.89 (0.81, 0.98)*	0.91 (0.83, 1.01)
Openness	-0.08 (-0.12, -0.04)***	-0.08 (-0.11, -0.04)***	0.96 (0.87, 1.06)	0.96 (0.87, 1.05)
**Political Orientation**	-0.03 (-0.07, 0.01)	-0.03 (-0.07, 0.01)	1.00 (0.91, 1.11)	1.00 (0.90, 1.12)
**Universalism * Funding Pressure**		0.01 (-0.03, 0.05)		0.92 (0.84, 1.02)
**Universalism * Publication Pressure**		-0.05 (-0.09, -0.01)*		0.96 (0.87, 1.06)
**Organised Scepticism * Funding Pressure**		0.01 (-0.04, 0.06)		1.00 (0.90, 1.11)
**Organised Scepticism * Publication Pressure**		-0.01 (-0.06, 0.04)		1.04 (0.95, 1.14)
**Communalism * Publication Pressure**		0.02 (-0.02, 0.06)		1.02 (0.93, 1.12)
**Communalism * Funding Pressure**		0.01 (-0.03, 0.05)		1.04 (0.94, 1.12)
**Conscientiousness * Publication Pressure**		0.04 (-0.00, 0.08)		1.02 (0.93, 1.12)
**Emotional Stability * Publication Pressure**		-0.04 (-0.08, 0.01)		0.91 (0.83, 1.00)*

**Openness * Publication Pressure**		0.00 (-0.03, 0.04)		0.99 (0.91, 1.09)
**Agreeableness * Publication Pressure**		-0.01 (-0.04, 0.02)		1.01 (0.92, 1.11)

n = 3016; Coefficients and confidence intervals (CIs) are based on pooled estimates across 15 imputed datasets, using Rubin's rules. CIs are bootstrapped. Sampling weights were included in all models. Respondents who completed six or fewer QRP items were excluded from the analysis using listwise deletion.

Overall mean QRP was computed based on 4-12 QRP items with not applicable responses removed.

Any frequent QRP is at least one of the 12 QRPs being often or always being engaged in.

All models contain the five background characteristics of sex, disciplinary field, methodological approach, career stage and country.

* p < .05; ** p < .01; *** p < .001.

Table S2.4. Regression Coefficients (95% CI) of proximal science-specific motivators (achievement motivation and impact and quality motivation) under MNAR-moderate assumption

	**Achievement Motivation**	**Impact and Quality Motivation**
	**Linear Regression Model 3A Coefficient (95% CI)**	**Linear Regression Model 3B Coefficient (95% CI)**
**Publication Pressure**	0.08 (0.04, 0.12)***	0.01 (-0.03, 0.05)
**Funding Pressure**	0.14 (0.10, 0.18)***	0.05 (0.00, 0.09)*
**Personal Motivations**		
Career Growth	0.33 (0.29, 0.37)***	0.05 (-0.00, 0.09)
Ego and Esteem	0.25 (0.22, 0.29)***	0.15 (0.11, 0.19)***
**Career Growth * Funding Pressure**	-0.03 (-0.07, 0.01)	0.00 (-0.04, 0.05)
**Career Growth * Publication Pressure**	0.03 (-0.01, 0.07)	0.00 (-0.04, 0.05)
**Ego and Esteem * Funding Pressure**	0.00 (-0.04, 0.04)	0.00 (-0.04, 0.05)
**Ego and Esteem * Publication Pressure**	-0.04 (-0.08, 0.01)	-0.04 (-0.09, 0.01)

n = 3050; Coefficients and confidence intervals (CIs) are based on pooled estimates across 15 imputed datasets, using Rubin's rules. CIs are bootstrapped. Respondents who completed six or fewer QRP items were excluded from the analysis using listwise deletion. Sampling weights were included in all models. Models include five background characteristics (sex, disciplinary field, methodological approach, career stage and country) and left-right political orientation scale. * p < 0.05; ** p < 0.01; *** p < 0.001.

Table S2.5. Path coefficients (95% CIs) and fit statistics for structural model of direct effects under MNAR-moderate assumption

	**Standardised Coefficient (95% CI)**
Career growth motivation (X1) → achievement motivation (M1)	0.39 (0.35, 0.43) ***
Ego and esteem motivation (X2) → achievement motivation (M1)	0.26 (0.23, 0.30) ***
Career growth motivation (X1) → Impact and quality motivation (M2)	0.06 (0.02, 0.11)**
Ego and esteem motivation (X2) → Impact and quality motivation (M2)	0.15 (0.11, 0.19) ***
Achievement motivation (M1) → mean QRP (Y)	0.10 (0.05, 0.14) ***
Impact and quality motivation (M2)→ mean QRP (Y)	-0.19 (-0.23, -0.15) ***
Career growth motivation (X1) → mean QRP (Y)	
Ego and esteem motivation (X2) → mean QRP (Y)	0.14 (0.10, 0.18) ***
R^2 Mean QRP	0.09 (0.00)
R^2 Achievement Motivation	0.34 (0.00)
R^2 Impact and Quality Motivation	0.07 (0.00)
Tucker-Lewis Index (TLI)	0.98
Root Mean Square Error of Approximation (RMSEA)	0.02
CFI (Comparative Fit Index)	0.99

n = 3016; Coefficients and SEs are based on pooled estimates across 15 imputed datasets, using Rubin's rules.

Sampling weights were included in all models. Respondents who completed six or fewer QRP items were excluded from the analysis using listwise deletion.

Non-significant demographic controls (*p* > 0.05) were removed and covariance between M1 and M2 was added, based on theoretical rationale and modification indices.

* p < 0.05; ** p < 0.01; *** p < 0.001.

Table S2.6. Path coefficients (95% CIs) for structural model of indirect effects under MNAR-moderate assumption

	Standardised Coefficient (95% CI)
Career growth motivation (X1) → achievement motivation (M1) → mean QRP (Y)	0.04 (0.02, 0.05) ***
Ego and esteem motivation (X2) → achievement motivation (M1) → mean QRP (Y)	0.03 (0.01, 0.04) ***
Career growth motivation (X1) → Impact and quality motivation (M2) → mean QRP (Y)	-0.01 (-0.02, -0.00)**
Ego and esteem motivation (X2) → Impact and quality motivation (M2) → mean QRP (Y)	-0.03 (-0.04, -0.02)***

n = 3016; Coefficients and SEs are based on pooled estimates across 15 imputed datasets, using Rubin's rules.

Sampling weights were included in all models. Respondents who completed six or fewer QRP items were excluded from the analysis using listwise deletion.

Non-significant demographic controls ($p > 0.05$) were removed and covariance between M1 and M2 was added, based on theoretical rationale and modification indices.

* p < 0.05; ** p < 0.01; *** p < 0.001.

S2.4. Mediation Analysis – SEM Modelling

Table S2.7. Fit indices for structural models of direct and indirect effects

	Model 4A	Model 4B	Model 4C (Final)
Tucker-Lewis Index (TLI)	-0.71	0.89	0.98
Root Mean Square Error of Approximation (RMSEA)	0.15	0.04	0.02
CFI (Comparative Fit Index)	0.96	0.96	0.99

n = 3050; Fit statistics were averaged across 15 imputed datasets.

Sampling weights were included in all models.

Model 1 included all five background characteristics (sex, disciplinary field, methodological approach, career stage, and country) as well as the left-right political orientation scale.

Model 4B removed non-significant demographic controls ($p > 0.05$). Model 4C added a covariance between M1 and M2, based on theoretical rationale and modification indices.

Table S2.8. Path coefficients (95% CIs) and fit statistics for structural model of direct effects

	SEM Model 4A Standardised Coefficient (95% CI / SE)	SEM Model 4B Standardised Coefficient (95% CI / SE)	Model 4C (Final) Standardised Coefficient (95% CI / SE)
Career growth motivation (X1) → achievement motivation (M1)	0.39 (0.35, 0.43)***	0.39 (0.36, 0.43) ***	0.39 (0.36, 0.43) ***
Ego and esteem motivation (X2) → achievement motivation (M1)	0.26 (0.23, 0.29) ***	0.26 (0.23, 0.30) ***	0.26 (0.23, 0.30) ***
Career growth motivation (X1) → Impact and quality motivation (M2)	0.06 (0.02, 0.11)**	0.06 (0.02, 0.11)*	0.07 (0.02, 0.10)**
Ego and esteem motivation (X2) → Impact and quality motivation (M2)	0.15 (0.11, 0.19) ***	0.15 (0.11, 0.19) ***	0.15 (0.11, 0.19) ***
Achievement motivation (M1) → mean QRP (Y)	0.10 (0.06, 0.15) ***	0.10 (0.06, 0.13) ***	0.10 (0.06, 0.13) ***
Impact and quality motivation (M2)→ mean QRP (Y)	-0.18 (-0.023, -0.14) ***	-0.19 (-0.23, -0.14) ***	-0.19 (-0.23, -0.15) ***
Career growth motivation (X1) → mean QRP (Y)	-0.02 (-0.06, 0.03)		
Ego and esteem motivation (X2) → mean QRP (Y)	0.15 (0.12, 0.19) ***	0.14 (0.10, 0.18) ***	0.14 (0.10, 0.18) ***
R^2 Mean QRP	0.09 (0.00)	0.09 (0.00)	0.09 (0.00)
R^2 Achievement Motivation	0.35 (0.00)	0.35 (0.00)	0.35 (0.00)
R^2 Impact and Quality Motivation	0.08 (0.00)	0.08 (0.00)	0.08 (0.00)

n = 3050; Coefficients and SEs are based on pooled estimates across 15 imputed datasets, using Rubin's rules.

Sampling weights were included in all models.

Model 1 included all five background characteristics (sex, disciplinary field, methodological approach, career stage, and country) as well as the left-right political orientation scale.

Model 2 removed non-significant demographic controls ($p > 0.05$). Model 3 added a covariance between M1 and M2, based on theoretical rationale and modification indices.

* p < 0.05; ** p < 0.01; *** p < 0.001.

Table S2.9. Path coefficients (95% CIs) and fit statistics for structural model of indirect effects

	**Model 4A Standardised Coefficient (SE)**	**Model 4B Standardised Coefficient (SE)**	**Model 4C (Final) Standardised Coefficient (SE)**
Career growth motivation (X1) → achievement motivation (M1) → mean QRP (Y)	0.04 (0.02, 0.06) ***	0.04 (0.02, 0.05) ***	0.04 (0.02, 0.05) ***
Ego and esteem motivation (X2) → achievement motivation (M1) → mean QRP (Y)	0.03 (0.01, 0.04) ***	0.03 (0.01, 0.04) ***	0.03 (0.01, 0.04) ***
Career growth motivation (X1) → Impact and quality motivation (M2) → mean QRP (Y)	-0.01 (-0.02, -0.00)*	-0.01 (-0.02, -0.00)**	-0.01 (-0.02, -0.00)**
Ego and esteem motivation (X2) → Impact and quality motivation (M2) → mean QRP (Y)	-0.03 (-0.04, -0.02) ***	-0.03 (-0.04, -0.02) ***	-0.03 (-0.04, -0.02)***

n = 3050; Coefficients and SEs are based on pooled estimates across 15 imputed datasets, using Rubin's rules.

Sampling weights were included in all models.

Model 1 included all five background characteristics (sex, disciplinary field, methodological approach, career stage, and country) as well as the left-right political orientation scale.

Model 2 removed non-significant demographic controls ($p > 0.05$). Model 3 added a covariance between M1 and M2, based on theoretical rationale and modification indices. * p < 0.05; ** p < 0.01; *** p < 0.001.
